# Super‐Enhancer‐Driven SOX4/SMAD3 Mediate Membrane Remodeling by Regulating Phospholipid Metabolism to Accelerate Leukemia Progression

**DOI:** 10.1002/advs.202512332

**Published:** 2026-02-21

**Authors:** Enzhe Lou, Peilong Lai, Guanjie Peng, Bo Lu, Lizhen Jiang, Xinyue Li, Jinghong Chen, Ladi Mo, Qiong Mao, Haichuan Zhang, Jinxin Fang, Yi Meng, Aochu Liu, Yingyin Gao, Andong Huang, Wenhui Lin, Shiwen Hu, Zerong Guan, Xiaolan Ye, Zhenguo Liu, Liling Jiang, Yueyuan Zheng, Xianping Shi

**Affiliations:** ^1^ Sino‐French Hoffmann Institute Guangzhou Municipal and Guangdong Provincial Key Laboratory of Protein Modification and Degradation School of Basic Medical Sciences Guangzhou Medical University Guangzhou Guangdong China; ^2^ The Affiliated Traditional Chinese Medicine Hospital Guangzhou Medical University Guangzhou Guangdong China; ^3^ Department of Hematology Guangdong Provincial People's Hospital (Guangdong Academy of Medical Sciences) Southern Medical University Guangzhou Guangdong China; ^4^ Clinical Big Data Research Center Scientific Research Center The Seventh Affiliated Hospital of Sun Yat‐Sen University Shenzhen Guangdong China; ^5^ Department of Hematology The Seventh Affiliated Hospital Sun Yat‑Sen University Shenzhen Guangdong China; ^6^ Laboratory Animal Center Guangzhou Municipal and Guangdong Provincial Key Laboratory of Protein Modification and Degradation School of Basic Medical Sciences Guangzhou Medical University Guangzhou Guangdong China; ^7^ Department of Thoracic Surgery The First Affiliated Hospital of Sun Yat‐Sen University Guangzhou Guangdong China

**Keywords:** blast phase, chronic myeloid leukemia, super enhancer, targeted therapies, transcription factor

## Abstract

Chronic myeloid leukemia (CML) is driven by the BCR‐ABL fusion oncogene and progresses from chronic phase (CP) to blast phase (BP). While tyrosine kinase inhibitors (TKIs) effectively control CML‐CP, CML‐BP remains a therapeutic challenge characterized by treatment resistance and poor survival. Accumulating evidence indicates that aberrant activation of epigenetic regulatory elements remodels the transcriptome in cancer, creating dependencies on specific transcriptional regulators that drive cancer progression. However, whether specific transcriptional mechanisms promote the transition from CML‐CP to CML‐BP remains unclear. This study identifies super‐enhancer‐driven transcription factors SOX4 and SMAD3 in CML‐BP. SOX4 and SMAD3 engage in a positive feedback axis through mutual binding to their respective super‐enhancers and promoters. Functional assays confirm that this axis promotes leukemic progression in vitro and in vivo. Mechanistically, SOX4/SMAD3 bind to the promoter and enhancer regions of the receptor tyrosine kinase AXL, enhancing its transcription and subsequently activating the AKT/ERK/STAT5 signaling. Concurrently, they transcriptionally upregulate LPCAT1, which remodels membrane phospholipids to facilitate AXL localization. Notably, the AXL inhibitor Bemcentinib effectively suppressed CML‐BP progression in both in vivo and in vitro models. Collectively, our findings establish SE‐driven SOX4 and SMAD3 as key regulators in CML‐BP and identify Bemcentinib as a promising therapeutic strategy.

## Graphical abstract

Chronic myeloid leukemia blast phase (CML‐BP) poses a severe therapeutic challenge. This study reveals that the super‐enhancer‐driven transcription factors SOX4 and SMAD3 form a cooperative axis critical for disease progression. They co‐activate the oncogenic kinase AXL and promote phospholipid remodeling via LPCAT1 to facilitate its signaling. Targeting this axis with Bemcentinib suppresses CML‐BP, highlighting its therapeutic promise.

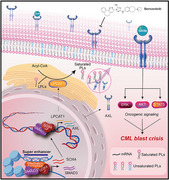

## 1 Introduction

Chronic myeloid leukemia (CML) is a clonal disorder of the pluripotent hematopoietic stem cell, in which a reciprocal translocation t(9;22)(q34;q11) creates a novel chimeric gene BCR‐ABL1 [1]. CML usually follows a triphasic course: chronic phase (CP), accelerated phase (AP), and blast phase (BP). While the CP is typically defined by the presence of <10% blasts in peripheral blood or bone marrow, the blast phase is characterized by≥20% blasts according to WHO criteria [2]. During blast phase, patients present with acute leukemia associated with poor clinical outcomes [3, 4]. Thus, understanding the molecular mechanisms of CML evolution and developing effective approaches to block or cure it remain unmet needs for patients.

Transcriptional dysregulation involves complex programs regulated by coordinated *cis*‐elements and *trans*‐factors [5]. Enhancers, the *cis*‐elements located at a long distance from their target promoters, play key roles in gene expression. Super‐enhancers (SEs) are clusters of multiple enhancers characterized by high level of bindings of H3K27ac, coactivators and transcription factors (TFs) [6]. Cancer‐associated SE domains mark lineage‐restricted genes and oncogenes, with higher expression than genes controlled by regular enhancers, indicating they may be acquired during tumorigenesis [7, 8]. For instance, our group revealed that SEs‐driven core transcriptional regulatory circuitry promotes Ewing's sarcoma occurrence and progression [9]. Additionally, SEs and their driver genes such as FOSL2, HAND2, MEIS2, etc., define neuroblastoma subtypes, and have reference significance for personalized therapy [10]. In leukemia, SEs also play a pivotal role in dysregulating key oncogenes. For instance, in T‐cell acute lymphoblastic leukemia (T‐ALL), SEs drive the expression of oncogenes like TAL1 and RUNX1 [11, 12], while in acute myeloid leukemia (AML), upon chromosomal rearrangement, the GATA2 distal hematopoietic enhancer acquires characteristics of a super‐enhancer and causes overexpression of EVI1 [13]. Based on this, since CML progresses through distinct clinical phases, unique SE domains and driver genes may contribute to the transformation of CML.

Here, we revealed the SEs‐driven transcription factors SOX4 and SMAD3, which can be self‐ and mutually transcriptionally activated by binding to their own and each other's promoters and enhancers, synergistically reshaping the transcriptional program in CML cells and accelerating blastic transformation. Specifically, SOX4/SMAD3 activates tyrosine kinase AXL‐mediated non‐BCR‐ABL‐dependent oncogenic signaling via two mechanisms, 1) directly binding to the promoter and enhancer of AXL for transcriptional activation, thereby upregulating AXL expression; 2) SOX4/SMAD3 enhance LPCAT1‐driven saturated phospholipid production to remodel the membrane, facilitating AXL membrane localization and activation. Meanwhile, we found that the AXL inhibitor Bemcentinib, which is in phase II clinical testing for non‐small cell lung cancer and myelodysplastic syndromes (NCT03824080, NCT02424617), can greatly alleviate the progression of CML. These findings suggest the potential of targeting the SOX4/SMAD3‐AXL axis in combination with standard therapy to block CML progression.

## 2 Results

### 2.1 Integrative Profiling Identifies SE‐Driven SOX4 and SMAD3 With Reciprocal Regulation in CML‐BP

**FIGURE 1:**
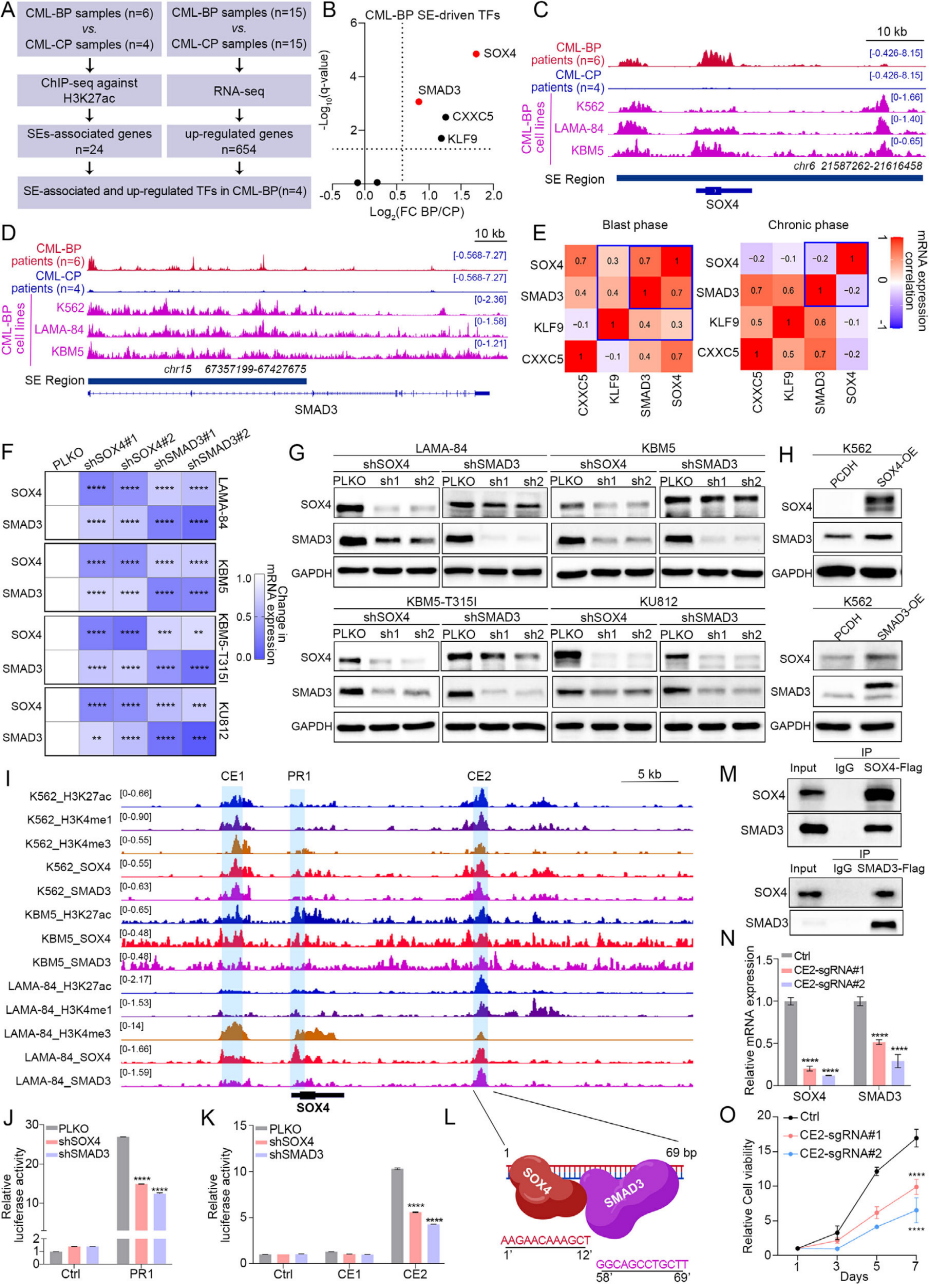
SEs‐driven transcription factors SOX4 and SMAD3 with Reciprocal Regulation in CML‐BP. (A) Schematic workflow for identifying super‐enhancer (SE)‐associated TFs in CML‐BP through integrated analysis of H3K27ac ChIP‐seq and RNA‐seq data from CML‐CP and CML‐BP patient samples. (B) mRNA expression levels of the four candidate SE‐driven TFs in CML‐CP versus CML‐BP from RNA‐seq (in‐house). (C‐D) IGV tracks of H3K27ac ChIP‐seq signals at the genomic loci of SOX4(C) and SMAD3(D) in CML‐CP patients, CML‐BP patients, and CML‐BP cell lines. (E) Pearson correlation analysis of candidate SE‐driven TFs across different stages of CML. (F) Heatmap depicting the fold changes in SOX4 and SMAD3 mRNA expression after individual knockdown of SOX4 or SMAD3 in the indicated CML‐BP cell lines. (G) Western blot analysis showing the protein levels of SOX4 and SMAD3 following individual knockdown with specific shRNAs in CML‐BP cell lines. (H) Western blot analysis of SOX4 and SMAD3 protein levels in K562 cells overexpressing SOX4 or SMAD3. (I) Representative IGV tracks showing co‐occupancy of SOX4 and SMAD3 at the SOX4 gene locus in CML‐BP cells. Two candidate enhancer regions (CE1, CE2) and one promoter region (PR1) are indicated. (J,K) Luciferase reporter assays measuring the activity of the SOX4 promoter (J) and the candidate enhancer (K) in LAMA‐84 cells upon knockdown of SOX4 or SMAD3. (L) Schematic diagram of the spatial configuration between SOX4 and SMAD3 binding motifs within the candidate enhancer CE2, as determined by HOMER motif analysis. (M) Co‐immunoprecipitation analysis of the interaction between SOX4 and SMAD3 in LAMA‐84 cells. (N) RT‐qPCR analysis of SOX4 and SMAD3 mRNA expression in LAMA‐84 cells after targeted epigenetic repression of the CE2 enhancer using a dCas9‐KRAB system. (O) The relative cell viability of LAMA‐84 cells after targeted epigenetic repression of the CE2 enhancer using a dCas9‐KRAB system. Data are presented as mean ± s.d. of three replicates. Two‐way ANOVA Dunnett's multiple comparisons test were performed. The experiments in panels (F–H), (J,K) and (M–O) were repeated three times independently with similar results, and the results of 1 representative experiment are shown. *p < 0.05, **p < 0.01, ***p < 0.001, ****p < 0.0001; ns, not significant. See also Figures S1 and S2.

Identifying the bona fide driving targets is crucial for investigating the mechanism of CML transformation and its clinical application. We first sought to identify genes by integrating the specific SE profiles and mRNA expression profiles of CML patients (Figure 1A). Briefly, we performed ChIP‐seq analysis of samples from CML‐CP (n = 4) and CML‐BP (n = 6) patients, using the antibody against H3K27ac. In parallel, we conducted RNA‐seq analysis on samples from CML‐CP (n = 15) and CML‐BP (n = 15) patients. Principal Component Analysis showed differences between CML‐BP and CML‐CP samples in ChIP‐seq and RNA‐seq (Figure S1A). SEs were identified from ChIP‐seq data of patient samples using the ROSE algorithm (Supplementary Table S1). Comparative analysis via DiffBind identified 24 BP‐specific SEs, which were then annotated to their closest genes (Supplementary Table S2). We then further identified four transcription factors (SMAD3, SOX4, CXXC5 and KLF9) that are upregulated in mRNA expression and driven by SEs in CML‐BP (Figure 1B). Integrative Genomics Viewer (IGV) demonstrated a marked elevation of H3K27ac signals at the enhancer regions of these TFs in CML‐BP (Figure 1C,D, S1B,C). Public RNA‐seq data showed that the expression of these TFs was also elevated in CML‐BP (Figure S1D).

Subsequent Pearson correlation analysis of the four TFs using RNA‐seq data from CML and AML (TCGA) patients revealed a strong positive correlation between SOX4 and SMAD3 specifically in CML‐BP, in contrast to their negative correlation in both CML‐CP and AML. (Figure 1E, S1E). To validate this strong correlation, and owing to the lack of established CML‐CP cell lines, we utilized cell lines derived from the CML‐BP for validation. Knockdown of SOX4 or SMAD3 alone reduced the mRNA and protein expression of another transcription factor in four CML‐BP cell lines (Figure 1F,G). On the contrary, exogenous overexpression of SOX4 or SMAD3 alone upregulated the protein level of the other (Figure 1H and S1F,G). Given this interdependence, we sought to target their SE‐driven expression therapeutically. Treatment with BET inhibitors (ABBV‐744, ARV‐771) resulted in a dose‐dependent suppression of both SOX4 and SMAD3 (Figure S1H), confirming their dependency on SE activity. Furthermore, motif analysis of their SE regions provided direct evidence of a reciprocal regulatory relationship between SOX4 and SMAD3. Both the SOX4 and SMAD3 genomic regions contain binding motifs for SMAD3 and SOX4 (Figure S1I). In summary, we identified the transcription factors SOX4 and SMAD3 as specifically super‐enhancer‐driven in CML‐BP, which engage in reciprocal regulation and represent potential therapeutic targets.

### 2.2 Mechanistic Dissection of the SOX4/SMAD3 Reciprocal Regulatory Axis

To elucidate the co‐regulatory mechanisms, we conducted CUT&Tag assays targeting SOX4 and SMAD3 in LAMA‐84, KBM5 and K562 cells. Importantly, the results showed two TFs dual‐occupied both enhancer and promoter of each other (Figure 1I and S1J). Furthermore, we performed ChIP‐re‐ChIP assays, which empirically confirmed the concurrent presence of both SOX4 and SMAD3 on the same DNA templates (Figure S2A), demonstrating their simultaneous occupation of these enhancer regions. Next, we identified one promoter region of SOX4 (PR1), and two candidate enhancer constituents (CE1, CE2) within the super‐enhancer of SOX4. These assumed regions and control regions were cloned into pGL3 luciferase reporter vector, and then transfected into LAMA‐84 and K562 cells. Robust reporter activities of PR1 and CE2, but not CE1, were observed (Figure 1J,K and S2B). We thus consider that CE1 harbors no activity. Then we silenced each TFs, and found that knockdown of each factor markedly reduced the activity of PR1 and CE2, overexpression results in vice versa (Figure 1J,K and S2B). These results support direct regulation of SOX4 promoter and super‐enhancer by SOX4 itself and its counterpart SMAD3. The spacing of TF binding sites can provide insights into the regulatory organization and cooperativity between TFs^14^. We thus performed HOMER motif analysis and observed that the spacing between the binding sites of SOX4 and SMAD3 within CE2 was less than 146 bps (Figure 1L). Additionally, the immunoprecipitation results showed that there was a protein interaction between SOX4 and SMAD3 (Figure 1M), supporting the tight spatial binding and functional cooperation between these TFs.

To further validate the activity and transcriptional functionality of CE2, we recruited a transcriptional repressor complex to this non‐coding region using a catalytically‐dead CRISPR‐Cas9 system (Figure S2C) [14]. This intervention resulted in a marked decrease in H3K27ac signal at this locus, indicating reduced chromatin accessibility (Figure S2D). Crucially, this led to diminished binding of both SOX4 and SMAD3 to this region (Figure S2D). Therefore, diminished enhancer activity at SOX4‐CE2 drives down the expression of its direct target, SOX4, at both the mRNA and protein levels (Figure 1N, S2E–G). This was accompanied by reduced expression of the reciprocal regulator SMAD3 (Figure 1N, S2G), ultimately impairing the proliferation of CML‐BP cells and promoting their erythroid and megakaryocytic differentiation (Figure 1O, S2H).

Next, we investigated the potential spatial basis underlying the transcriptional regulation of SOX4 by SOX4‐CE2 by chromosome conformation capture (3C) assay (Figure S2I). The results demonstrated that the frequency of contacts between SOX4‐CE2 and promoter was significantly higher compared to Control fragments (Figure S2J,K). 3C assay followed by Sanger sequencing confirmed chromosomal interactions between the SOX4 promoter and SOX4‐CE2, with both SOX4 and SMAD3 motifs identified in the interacting regions (Figure S2L). These results collectively indicate that there is a direct spatially physical connection between the SE constituent SOX4‐CE2 and the SOX4 promoter, which provides the possibility for regulating the transcription of SOX4.

### 2.3 SOX4/SMAD3 Cooperatively Orchestrate the Transcriptional Network in CML Progression

**FIGURE 2:**
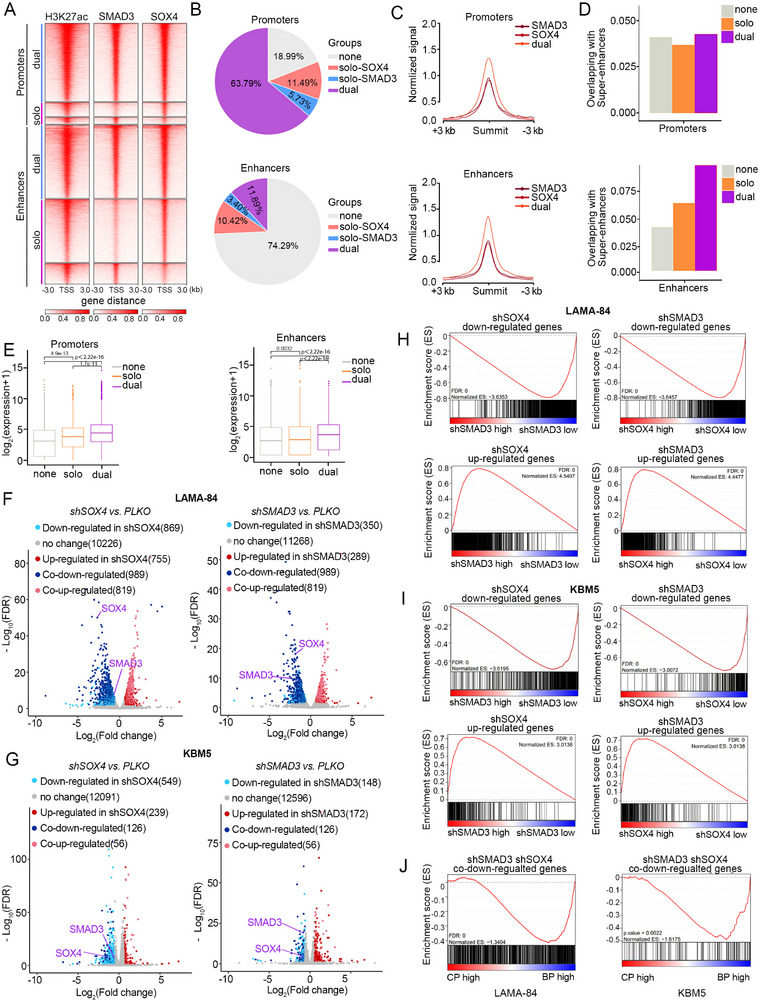
SOX4/SMAD3 cooperatively orchestrate the transcriptional network in CML progression. (A) Heatmaps displaying CUT&Tag signals for SOX4, SMAD3, and H3K27ac in LAMA‐84 cells, centered on peak summits and grouped by distinct SOX4/SMAD3 co‐binding patterns (SOX4‐only, SMAD3‐only, dual‐bound). (B) Pie chart quantification of the genome‐wide distribution of SOX4 and SMAD3 binding patterns in LAMA‐84 cells, showing the relative proportions of solo‐ and co‐bound regions. (C) Line plots of H3K27ac CUT & Tag signals from indicated groups of peaks in LAMA‐84 cells. (D) The overlapping of indicated groups of peaks with super‐enhancers in LAMA‐84 cells. (E) Box plots of mRNA expression of genes regulated by the indicated groups of peaks in LAMA‐84 cells. (F,G) Volcano plots of differentially expressed genes from RNA‐seq following individual knockdown of SOX4 or SMAD3 in LAMA‐84 (F) and KBM5 (G) cells. Significantly up‐ and down‐regulated genes are highlighted. (H‐I) GSEA plots showing the enrichment of regulated genes upon knockdown of SOX4/SMAD3 in RNA‐Seq data of LAMA‐84(H) and KBM5(I) cells. The top panels show enrichment for downregulated genes, and the bottom panels for upregulated genes upon knockdown. NES, normalized enrichment score; FDR, false discovery rate. (J) GSEA plots validating the significant enrichment of the co‐downregulated genes by knocking down SOX4 or SMAD3 within “blast phase” transcriptional signature derived from patient RNA‐seq data (CML‐BP vs. CML‐CP, in‐house). Analysis was performed for genes co‐downregulated in both LAMA‐84 (left) and KBM5 (right) cell lines.

To investigate the mechanistic foundation of SOX4 and SMAD3 in CML transcriptome regulation, we analyzed the epigenomic characteristics of their occupancy in LAMA‐84 and K562 cells. To this end, we initially annotated the promoter and enhancer regions using available histone modification, followed by genome‐wide peaks of SOX4/SMAD3 assigned to these regions (Figure 2A, S3A). Notably, in LAMA‐84 and K562 cells, SOX4/SMAD3 demonstrated cooperative occupancy (dual‐binding by SOX4 and SMAD3) than solo‐occupancy in both the promoter and enhancer, suggesting strong cooperativity between SOX4 and SMAD3 (Figure 2B and S3B). Importantly, regardless of whether the promoter or enhancer elements, dual‐binding regions exhibited much higher H3K27ac intensity compared to solo‐binding regions, suggesting that TFs co‐occupied regions likely have stronger transcriptional activity (Figure 2C and S3C). Moreover, dual‐bound regions were more likely to overlap super‐enhancers than solo‐binding regions (Figure 2D and S3D). To determine further the transcriptional impact of these binding events, matched RNA‐Seq data of LAMA‐84 and K562 cells were analyzed. Notably, transcriptional profiling showed that genes associated with dual‐bound promoters or enhancers exhibited significantly higher expression levels than solo‐bound promoters or enhancers (Figure 2E and S3E), supporting synergistic transcriptional regulation of SOX4 and SMAD3. To further elucidate the molecular basis of this cooperativity, we analyzed the spatial relationship of SOX4 and SMAD3 binding motifs within their dual‐bound peaks (Figure 2B, S3B). Notably, in approximately 40% of regions containing both motifs, the binding sites were situated within a nucleosome‐spanning distance (<146 bp) (Figure S3F,G), suggesting that genomic proximity facilitates their cooperative binding. We next assessed the interdependence of their chromatin occupancy. CUT&Tag in LAMA‐84 cells revealed that knockdown of either factor globally reduced binding of both partners, accompanied by diminished H3K27ac signals, indicating loss of chromatin accessibility (Figure S3H,I). IGV analysis confirmed a reciprocal reduction in SOX4 and SMAD3 binding at their promoters and enhancers following the knockdown of either factor (Figure S4A). Collectively, SOX4 and SMAD3 constitute a cooperative leukemogenic module, functionally interdependent through coordinated chromatin binding.

Having established this cooperative module, we functionally defined its transcriptomic landscape by performing RNA‐seq in LAMA‐84 and KBM5 cells following individual knockdown of SOX4 or SMAD3 (Figure 2F,G). Gene set enrichment analyses (GSEA) showed that genes decreased or increased following silencing of SOX4 were significantly enriched in those also downregulated or upregulated upon depletion of SMAD3, and vice versa (Figure 2H,I). The genes that were reduced after the individual silencing of SOX4 or SMAD3, as well as genes co‐downregulated, were enriched in the “blast phase” related gene set (in‐house and EGAS00001003071) (Figure 2J and S4B). Our findings establish SOX4/SMAD3 cooperativity as a mechanistic feature of transcriptional dysregulation in CML progression, with particular relevance to blast crisis transformation.

### 2.4 Loss of SOX4/SMAD3 Impairs CML Progression In Vitro

**FIGURE 3:**
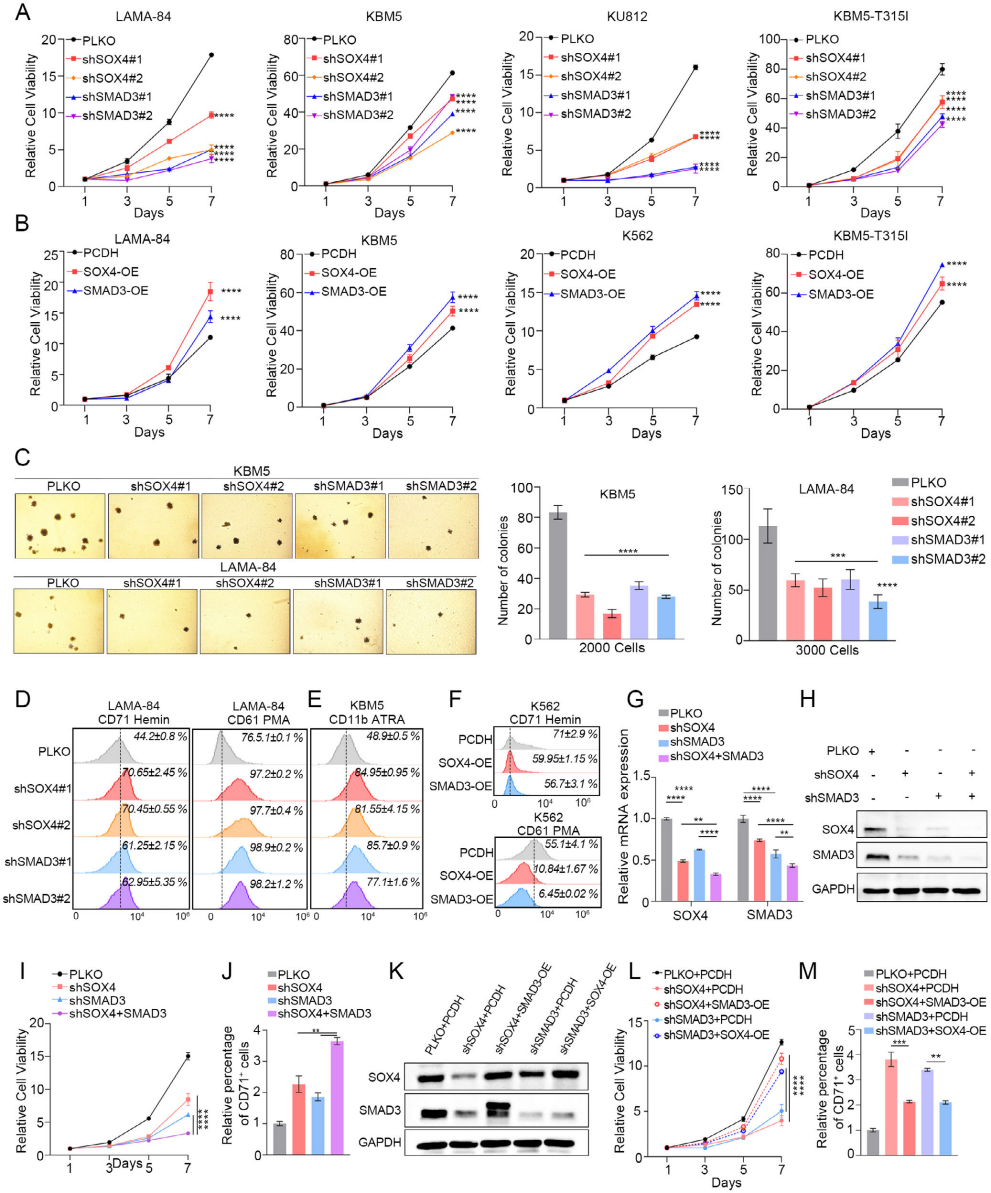
Loss of SOX4/SMAD3 impairs CML progression in vitro. (A) The relative cell viability of CML‐BP cell lines following stable knockdown of SOX4 or SMAD3 with specific shRNAs. (B) The relative cell viability of CML‐BP cells after ectopic overexpression of SOX4 or SMAD3 cDNA constructs. (C) Soft agar colony formation assay in KBM5 and LAMA‐84 cells with indicated knockdown. Left panel: Representative micrographs of colonies. Right panel: Quantitative analysis of colony numbers. (D) Flow cytometry analysis of erythroid differentiation marker CD71 (left panel) and megakaryocytic differentiation marker CD61 (right panel) in LAMA‐84 cells with SOX4 or SMAD3 knockdown following induction with Hemin (erythroid) or PMA (megakaryocytic). (E) Flow cytometry analysis of granulocytic differentiation marker CD11b in KBM5 cells with SOX4 or SMAD3 knockdown following treatment with all‐trans retinoic acid (ATRA). (F) Flow cytometry analysis of CD71 (Top) and CD61 (Bottom) surface expression in K562 cells overexpressing SOX4 or SMAD3 cDNA constructs. (G,H) Validation of dual knockdown efficiency. (G) RT‐qPCR and (H) Western blot analyses confirming the coordinated reduction of both SOX4 and SMAD3 at mRNA and protein levels in LAMA‐84 cells with simultaneous knockdown of both transcription factors. (I‐J) Functional consequences of dual knockdown. (I) Cell viability assay and (J) Flow cytometry analysis of CD71 surface expression in LAMA‐84 cells following dual knockdown of SOX4 and SMAD3. (K) Western blot verification of SOX4 and SMAD3 protein levels in LAMA‐84 cells with knockdown of one TF and concurrent overexpression of the other. (L) The relative cell viability of LAMA‐84 cells with knockdown of one TF and concurrent overexpression of the other.(M) Representative flow cytometry histograms showing CD71 surface expression in LAMA‐84 cells from the corresponding rescue groups. Data are presented as mean ± s.d. of three replicates. Two‐way ANOVA Dunnett's multiple comparisons test were performed. The experiments in panels (A–M) were repeated three times independently with similar results, and the results of 1 representative experiment are shown. *p < 0.05, **p < 0.01, ***p < 0.001, ****p < 0.0001; ns, not significant.

We performed biological function assays to validate whether the blast phase transformation of CML cells is dependent on SOX4 and SMAD3 expression. First, we found that in all three BCR‐ABL CML‐BP cell lines (LAMA‐84, KBM5, KU812) and one BCR‐ABL^T315I^ CML‐BP cell line (KBM5‐T315I), shSOX4 or shSMAD3 expressing cells exhibited slow proliferation rate (Figure 3A). On the contrary, exogenous overexpression of SOX4/SMAD3 enhanced the proliferation ability of CML cells (Figure 3B). Importantly, SOX4/SMAD3 knockdown resulted in significantly smaller colonies and 40∼60% reduction in the colony‐forming ability (Figure 3C). On the other hand, silencing or overexpressing SOX4/SMAD3 affected the differentiation ratios in different CML cell lines. Knockdown of SOX4 or SMAD3 promoted megakaryocyte differentiation (CD61) and erythroid differentiation (CD71) in LAMA‐84 cells (Figure 3D). Meanwhile, All‐trans‐retinoic acid (ATRA) stimulation enhanced granulocyte differentiation (CD11b) in KBM5 cells with TFs knockdown (Figure 3E). In addition, overexpression of SOX4/SMAD3 in K562 cells reduced the proportion of cells positive for megakaryocyte and erythroid differentiation (Figure 3F).

To clarify the synergistic biological functions of SOX4 and SMAD3 in the progression of CML, we constructed cells with SOX4 and SMAD3 co‐knockdown (Figure 3G,H). The proliferation and differentiation ability of CML cells TFs co‐knockdown was further affected compared to CML cells knockdown alone (Figure 3I,J). In addition, overexpression of the counterpart transcription factor could partially restore the acute properties after silencing, that is, overexpression of SMAD3 can restore the reduced proliferation and blastic transformation abilities of knocked down SOX4, and vice versa (Figure 3K–M). In summary, SOX4/SMAD3 synergistically promote proliferation and inhibit differentiation, thereby promoting the progression of CML.

### 2.5 SOX4/SMAD3 Co‐Regulate AXL Pathway in CML Progression

**FIGURE 4:**
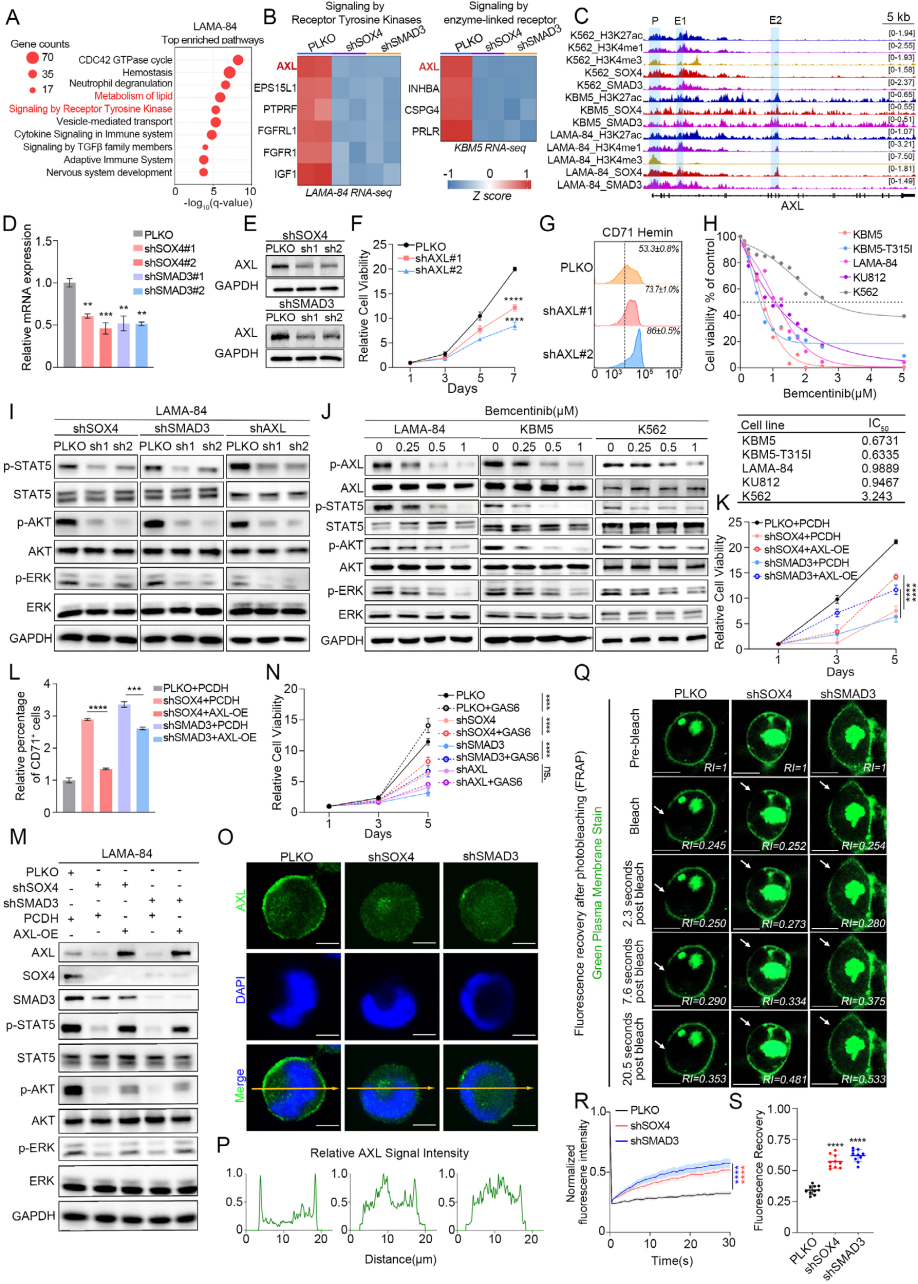
SOX4/SMAD3 co‐regulate AXL pathway in CML progression. (A) Reactome pathway enrichment analysis of the shared downregulated genes following knockdown of SOX4 or SMAD3 in LAMA‐84 cells. (B) Heatmaps show the mRNA expression of the down‐regulated receptor genes in “Signaling by Receptor Tyrosine Kinases (LAMA‐84)” and “Signaling by enzyme‐linked receptor (KBM5) ”. (C) IGV tracks showing simultaneous binding of SOX4 and SMAD3 at the AXL gene locus in CML‐BP cell lines, with specific binding at promoter and enhancer regions indicated. (D,E) RT‐qPCR (D) and Western blot (E) analyses of AXL mRNA and protein expression in LAMA‐84 cells following SOX4 or SMAD3 knockdown. (F) The relative cell viability of LAMA‐84 cells expressing the empty vector or shAXL.(G) Flow cytometry analysis of CD71 expression in LAMA‐84 cells following AXL knockdown. (H) Dose‐response curves measuring cell viability of indicated CML‐BP cell lines treated with the AXL inhibitor Bemcentinib for 48 h (I) Western blot analysis of AKT, STAT5, and ERK phosphorylation following knockdown of SOX4, SMAD3, or AXL in LAMA‐84 cells. (J) Western blot analysis of signaling pathway activation in LAMA‐84, KBM5 and K562 cells treated with Bemcentinib. (K) Cell viability assay in SOX4‐ or SMAD3‐deficient LAMA‐84 cells following AXL overexpression. (L) Representative flow cytometry histogram of surface CD71 expression in shSOX4‐ or shSMAD3‐transduced LAMA‐84 cells, following overexpression of AXL cDNA construct. (M) Western blot verification of signaling pathway restoration in rescue experiments. (N) The relative cell viability of LAMA‐84 cells stimulated with recombinant human GAS6 (500 ng/mL). (O) Immunofluorescence staining show the AXL localization (green) in LAMA‐84 cells with indicated shRNA or vector. Nuclei were stained with DAPI (blue). Scale bar, 5 µm. (P) Quantitative intensity profiles of AXL membrane localization along the indicated yellow line in (O). (Q) Representative FRAP images taken over a 30‐second time course in control and knockdown cells. Arrows indicate photobleaching sites. Scale bar, 10 µm. (R) FRAP recovery curves following treatment with indicated shRNA or vector in LAMA‐84 cells. n = 10. (S) Quantification of total fluorescence recovery following photobleaching after treatment with indicated shRNA or vector in LAMA‐84 cells. n = 10. Data are presented as mean ± s.d. of three replicates. Two‐way ANOVA Dunnett's multiple comparisons test were performed. The experiments in panels (D‐S) were repeated three times independently with similar results, and the results of 1 representative experiment are shown. *p < 0.05, **p < 0.01, ***p < 0.001, ****p < 0.0001; ns, not significant. See also Figure S5.

Next, we focused on the downstream pathways regulated by SOX4/SMAD3. Pathway enrichment analysis was performed using shared downregulated genes upon knockdown of each TF individually in LAMA‐84 and KBM5 cells (Figure S4C). In the two CML cell lines, several shared or interrelated pathways were observed (Figure 4A, S5A), including but not limited to “Signaling by Receptor Tyrosine Kinases” and its affiliated “Signaling by enzyme‐linked receptor,” along with “lipid metabolism.” These overlapping regulatory networks suggest potential functional convergence between the two cell models. Our initial focus was directed toward “Signaling by Receptor Tyrosine Kinases” and “Signaling by enzyme‐linked receptor,” given that occurrence of CML is driven by the tyrosine kinase BCR‐ABL, but currently there are other independent tyrosine kinases that play a role in malignant progression [15, 16]. Given the pivotal role of BCR‐ABL in CML, we first verified its involvement in SOX4/SMAD3‐driven CML advancement. Knockdown of SOX4/SMAD3 showed no alteration in BCR‐ABL mRNA or protein levels (Figure S5B,C), suggesting potential regulation of alternative tyrosine kinase signaling pathways by SOX4/SMAD3.

By comprehensively analyzing receptor expression changes across both the “Receptor Tyrosine Kinases” and “Enzyme‐Linked Receptor” signaling pathways, we systematically established AXL as a common downstream receptor (Figure 4B). AXL, which was first identified in CML and has been reported to be associated with drug resistance in CML [17, 18, 19]. SOX4/SMAD3 mainly bind to the promoter and enhancer elements of AXL (Figure 4C, S5D,E). Silencing either SOX4 or SMAD3 inhibited the expression of AXL as validated by RT‐qPCR and Western blot (Figure 4D,E and S5F,G). To define the role of AXL, we transduced the CML cell lines with control shRNA or AXL shRNA lentivirus (Figure S5H,I), the results showed AXL knockdown impaired the proliferation and promoted the differentiation (Figure 4F,G and S5J,K). Similarly, Bemcentinib, a selective molecule AXL inhibitor [20], could effectively inhibit the proliferation of CML cells and promote differentiation (Figure 4H, S5L). Moreover, immunoblotting assays confirmed that SOX4, SMAD3 or AXL knockdown genetically, as well as pharmacological inhibition of AXL with Bemcentinib, all reduced phosphorylation levels of STAT5, AKT and ERK in CML cells (Figure 4I,J and S5M). Notably, AXL similarly does not regulate the expression or phosphorylation of BCR‐ABL (Figure S5N–O), suggesting that SOX4/SMAD3 may promote CML progression through AXL‐mediated activation of BCR‐ABL‐independent oncogenic pathways. We then evaluated the synergistic effect of imatinib and Bemcentinib using SynergyFinder tool and obtained a strong synergy between two drugs (Figure S5P). Next, we attempted to rescue the shSOX4 or shSMAD3 proliferation phenotype with cDNA expression of AXL. As expected, we found that AXL rescued the growth defect of CML cells expressing shSOX4 or shSMAD3, impeding cell differentiation, and activating the phosphorylation levels of several central mediators (Figures 4K–M). As a membrane receptor, the function of AXL mainly relies on binding to the ligand GAS6 [21]. We first confirmed that knockdown of SOX4, SMAD3, or AXL did not affect endogenous GAS6 expression (Figure S5Q). Then, the addition of exogenous GAS6 only slightly rescued the proliferative phenotype of SOX4/SMAD3 knockdown cells, but had no effect on AXL knockdown cells (Figure 4N), which may be due to the extremely low expression of AXL. Consistently, the activation of downstream oncogenic signaling pathways followed a trend similar to the proliferative phenotype (Figure S5R).

As previously described, the function of AXL depends on its membrane localization and ligand binding, and we conducted simultaneously immunofluorescence analysis on TFs‐knockdown cells as well. Surprisingly, after silencing SOX4 and SMAD3, immunofluorescence showed the membrane localization of AXL was reduced (Figure 4O,P and S5S). Plasma membrane localization of AXL has been shown critical for receptor activity and signal transduction. The signaling activity of membrane receptors is influenced by the biophysical properties of the membrane, including curvature, charge, fluidity, and local structure. After labeling the cell membranes of living cells with fluorescent dyes, fluorescence recovery after photobleaching (FRAP) experiments revealed that SOX4/SMAD3 knockdown resulted in an increase in cell membrane fluidity (Figure 4Q–S).

Taken together, these data demonstrate that SOX4/SMAD3 coordinately promote the transcription of AXL, and regulate its membrane localization by mediating membrane fluidity, thereby activating oncogenic signaling cascades in CML. However, it remains unknown how SOX4/SMAD3 regulate cell membrane fluidity.

### 2.6 SOX4/SMAD3 regulated Saturated Phospholipid Synthesis to Mediate AXL Localization and Activation

**FIGURE 5:**
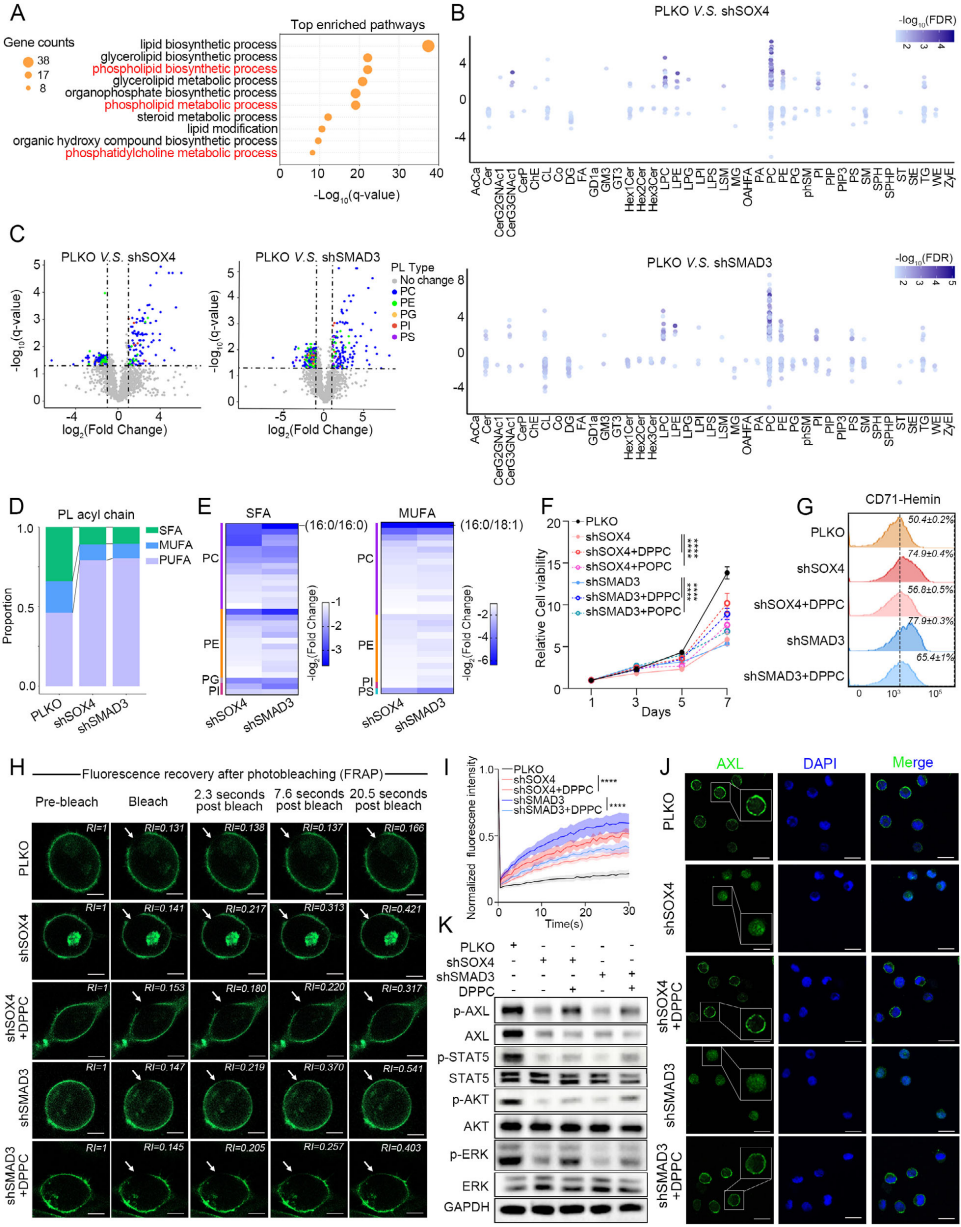
SOX4/SMAD3 regulated saturated phospholipid synthesis to mediate AXL localization and activation. (A) GO pathway enrichment analysis of the downregulated lipid metabolism‐associated genes following knockdown of SOX4 and SMAD3 in LAMA‐84 cells. (B) Distribution of changes in each subclass of the lipid metabolome upon knockdown of SOX4 or SMAD3 in LAMA‐84 cells. (C) Volcano plots showing differentially expressed phospholipids upon knockdown of SOX4 or SMAD3. Each dot represents a phospholipid. (D) Repartition of PL‐SFAs, PL‐MUFAs and PL‐PUFAs in LAMA‐84 cells transfected with shRNA targeting SOX4, SMAD3, or vector control. (E) Heatmap visualization of specific saturated and monounsaturated phospholipid species altered in LAMA‐84 cells expressing indicated shRNA or vector, highlighting the marked reduction of PC(16:0/16:0) (DPPC) and PC(16:0/18:1) (POPC). (F) The relative cell viability of SOX4/SMAD3‐deficient LAMA‐84 cells following treatment with DPPC or POPC liposomes. (G) Representative flow cytometry histogram of surface CD71 expression in LAMA‐84 cells expressing empty vector and SOX4/SMAD3 shRNA. Cells were treated with vehicle control or DPPC liposomes. (H) FRAP images in LAMA‐84 cells transfected with shRNA targeting SOX4, SMAD3, or empty vector. Cells were treated with vehicle control or DPPC liposomes. Images are taken over a 30 s time course and arrows indicate location of photobleaching. n = 10. Scale bar, 5 µm. (I) FRAP recovery curves following treatment with SOX4/SMAD3 shRNA or vector in LAMA‐84 cells. Cells were treated with vehicle control or DPPC liposomes. n = 10. (J) Immunofluorescence staining of AXL localization (green) in LAMA‐84 cells with SOX4/SMAD3 shRNA or vector. DPPC liposomes was added to rescue membrane localization. Nuclei were stained with DAPI (blue). Scale bar, 20 µm. (K) Western blot analysis of the total and phosphorylation levels of AKT, STAT5 and ERK upon knockdown of SOX4 or SMAD3 in LAMA‐84 cells. Cells were treated or untreated with DPPC. Data are presented as mean ± s.d. of three replicates. Two‐way ANOVA Dunnett's multiple comparisons test were performed. The experiments in panels (F–K) were repeated three times independently with similar results, and the results of 1 representative experiment are shown. *p < 0.05, **p < 0.01, ***p < 0.001, ****p < 0.0001; ns, not significant. See also Figure S6.

As shown in the previous analysis, SOX4/SMAD3 regulates the lipid metabolism pathway (Figure 4A). Corroborating this, GSEA revealed significant enrichment of lipid metabolic gene sets in CML‐BP (Figure S6A), suggesting that dysregulated lipid metabolism may represent a potential feature of blast crisis progression. To elucidate the mechanisms underlying the regulation of lipid metabolism, we analyzed in‐depth the enriched genes in the term ‘Metabolism of lipids’. These genes were enriched in key pathways in phospholipid metabolism and specific phosphatidylcholine metabolism (Figure 5A). Phospholipids (PL), particularly phosphatidylcholine (PC), are major components of the cell membrane, and their saturation levels are closely linked to membrane order and fluidity [22]. To quantify the impact of SOX4/SMAD3 on lipid metabolism, we performed untargeted lipidomics in LAMA‐84 cells, and identified 3314 lipid species belonging to 41 lipid classes (Figure 5B and S6B). Consistent with the pathway enrichment analysis, silencing SOX4 or SMAD3 caused appreciable changes in PL landscape, manifested as the proportion of PL containing saturated fatty acid (SFA) and monounsaturated fatty acids (MUFA) was significantly decreased, while the proportion of PL containing polyunsaturated fatty acids (PUFA) was increased (Figure 5C,D). In order to determine whether SFA‐PL or MUFA‐PL mainly mediated the phenotypic effects of SOX4/SMAD3, we selected the DPPC (16:0/16:0) (SFA‐PL) and POPC (16:0/18:1) (MUFA‐PL), which were the most obvious changes after SOX4/SMAD3 knockdown (Figure 5E), for rescue experiments. Proliferation assays showed that exogenous DPPC could more significantly rescue the proliferation defects caused by SOX4/SMAD3 knockdown (Figure 5F). Consistently, DPPC was able to partially rescue the differentiation (Figure 5G and S6C). To investigate whether the mechanisms underlying these phenotypic reversals result from DPPC‐induced alterations in membrane fluidity following plasma membrane remodeling, we conducted FRAP experiments. Upon exogenous addition of DPPC and their subsequent incorporation into the plasma membrane, the membrane fluidity was restored in CML cells with SOX4 and SMAD3 knockdown (Figure 5H,I and S6D), consequently recovering AXL localization and activation (Figure 5J,K). This suggests that SOX4/SMAD3 not only directly regulate AXL expression but also mediate its distribution through modulating lipid metabolism.

### 2.7 SOX4/SMAD3 Regulate LPCAT1 to Mediate Plasma Membrane Remodeling

**FIGURE 6:**
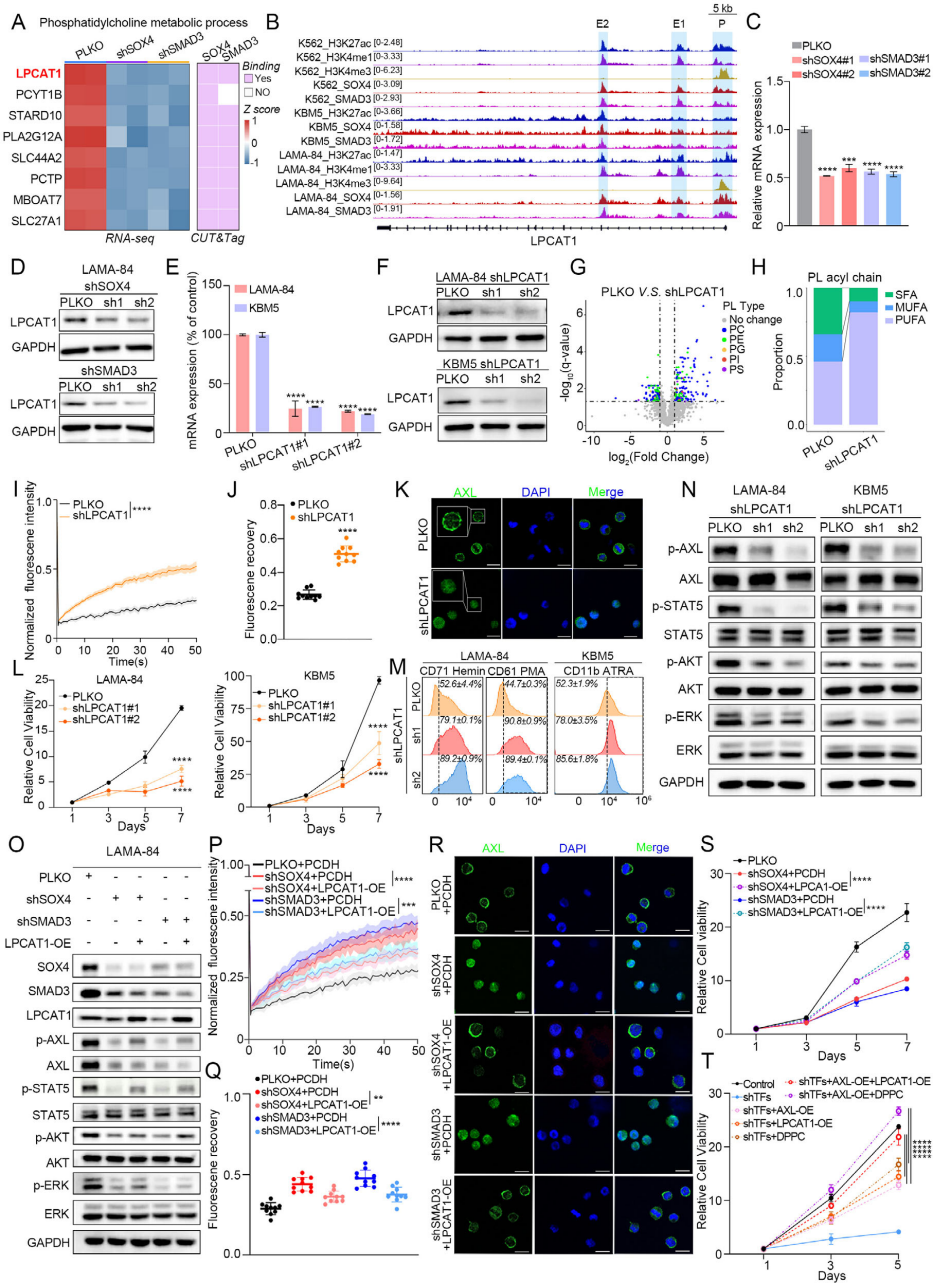
SOX4/SMAD3 regulate LPCAT1 to mediate plasma membrane remodeling. (A) Heatmap showing the mRNA expression of down‐regulated genes in “Phosphatidylcholine metabolic process pathway” in LAMA‐84 cells following SOX4 or SMAD3 knockdown (left panel). Heatmap displaying SOX4 and SMAD3 binding patterns at regulatory regions of these genes (right panel). (B) Representative IGV tracks showing simultaneous binding of SOX4 and SMAD3 at the LPCAT1 gene locus in CML‐BP cell lines. (C) RT‐qPCR analysis of LPCAT1 mRNA expression in LAMA‐84 cells transfected with shRNA targeting SOX4, SMAD3, or empty vector control. (D) Western blot analysis of LPCAT1 protein expression in LAMA‐84 cells following SOX4 or SMAD3 knockdown. (E) Knockdown efficiency validation of LPCAT1 shRNAs at mRNA level in KBM5 and LAMA‐84 cells by RT‐qPCR analysis. (F) Western blot analysis of LPCAT1 protein expression in KBM5 and LAMA‐84 cells following LPCAT1 knockdown. (G) Volcano plots identifying significantly altered phospholipid species upon knockdown of LPCAT1 in LAMA‐84 cells. Each dot represents an individual phospholipid species. (H) Repartition of PL‐SFAs, PL‐MUFAs and PL‐PUFAs in LAMA‐84 cells expressing empty vector and LPCAT1 shRNA. (I) FRAP recovery curves following photobleaching in LAMA‐84 cells transfected with shRNA targeting LPCAT1 or control. n = 10. (J) Quantification of total fluorescence recovery following photobleaching in LAMA‐84 cells after LPCAT1 knockdown. n = 10. (K) Immunofluorescence staining of AXL localization (green) in LAMA‐84 cells with LPCAT1 shRNA or vector. Nuclei were stained with DAPI (blue). Scale bar, 20 µm. (L) The relative cell viability of LAMA‐84 and KBM5 cells with shLPCAT1 or empty vector. (M) Representative flow cytometry histogram of surface CD71 and CD61 expression in LAMA‐84 cells with shLPCAT1 or empty vector (Left panel). And the surface CD11b expression in KBM5 cells with shLPCAT1 or empty vector (Right panel) (N) Western blot analysis of total and phosphorylation levels of AKT, STAT5 and ERK in LAMA‐84 and KBM5 cells following LPCAT1 knockdown. (O) Western blot analysis of the total and phosphorylation levels of indicated protein in shSOX4‐ or shSMAD3‐transduced LAMA‐84 cells, following overexpression of LPCAT1 cDNA construct. (P) FRAP recovery curves following photobleaching in SOX4/SMAD3‐deficient LAMA‐84 cells rescued by LPCAT1 overexpression. n = 10. (Q) Quantification of total fluorescence recovery following photobleaching after overexpression of LPCAT1 cDNA construct to rescue membrane fluidity after SOX4/SMAD3 depletion. n = 10. (R) Immunofluorescence staining of AXL localization (green) in SOX4/SMAD3‐deficient LAMA‐84 cells rescued by LPCAT1 overexpression. Nuclei were stained with DAPI (blue). Scale bar, 20 µm. (S) The relative cell viability of LAMA‐84 cells with indicated treatments. (T) The relative cell viability of SOX4/SMAD3‐deficient LAMA‐84 cells (shTFs) rescued by AXL or LPCAT1 overexpression, DPPC supplementation, or their combinations. Data are presented as mean ± s.d. of three replicates. Two‐way ANOVA Dunnett's multiple comparisons test were performed. The experiments in panels (C–F), and (I‐T) were repeated three times independently with similar results, and the results of 1 representative experiment are shown. *p < 0.05, **p < 0.01, ***p < 0.001, ****p < 0.0001; ns, not significant. See also Figure S6.

Next, we considered how SOX4/SMAD3 regulates SFA‐PL metabolism, thereby affecting membrane structure and fluidity, and thus regulating AXL localization and activation. Combining RNA‐seq and CUT&Tag, we focused on LPCAT1, which is a key enzyme in the membrane lipid remodeling (Figure 6A). SOX4 and SMAD3 direct bind to the promoter and enhancer elements of LPCAT1 (Figure 6B and S6E,F). Additionally, LPCAT1 expression was upregulated in CML‐BP patients (Figure S6G). The mRNA and protein expression of LPCAT1 was inhibited following the individual knockdown of SOX4 and SMAD3 (Figure 6C,D and S6H,I). To confirm the effect of LPCAT1, we silenced it using shRNA (Figure 6E,F), which was consistent with the effects of SOX4/SMAD3 knockdown. This also led to changes in lipid content, especially phospholipids, and reduced the degree of membrane phospholipid saturation (Figure 6G,H and S6J). Additionally, the content of DPPC was also greatly reduced in shLPCAT1 cells (Figure S6K). Remarkably, LPCAT1 depletion altered membrane order and fluidity, thereby promoting AXL internalization from the plasma membrane to intracellular compartments (Figure 6I,K). Furthermore, LPCAT1 depletion attenuated cell proliferation, promoted differentiation, and reduced AXL‐mediated downstream signaling activation (Figure 6L–N). To clarify the important role of LPCAT1‐mediated membrane phospholipid remodeling as a downstream mediator of SOX4/SMAD3, we attempted to rescue the proliferative phenotypes of shSOX4 or shSMAD3 by expressing LPCAT1 cDNA (Figure 6O). Indeed, overexpression of LPCAT1 restored membrane fluidity, proliferation, AXL membrane localization and activation (Figure 6O–S). Additionally, the LPCAT1 knockdown phenotype was partially restored by either DPPC supplementation or AXL overexpression (Figure S6L,M). Finally, to investigate whether SOX4/SMAD3 promotes CML progression primarily through AXL transcriptional regulation or via LPCAT1‐mediated membrane remodeling facilitating AXL localization and activation, experiments revealed neither AXL/LPCAT1 overexpression nor DPPC supplementation alone completely reversed the proliferative. Only combined intervention fully restored phenotypes, indicating dual regulatory mechanisms (Figure 6T and S6N). Taken together, transcription factors SOX4 and SMAD3 can affect membrane dynamics by regulating LPCAT1, thereby regulate membrane localization and activation of AXL, and ultimately promote the progression of CML.

### 2.8 Targeting SOX4/SMAD3‐AXL Axis Suppresses CML Progression In Vivo

To examine the in vivo roles of SOX4 and SMAD3 in CML progression, we employed two well‐established mouse models, both of which have been implicated in CML‐BP pathogenesis: one driven by BCR‐ABL alone (BA CML mice) and another co‐expressing BCR‐ABL with NUP98‐HOXA9 (BA/NH CML mice) [23, 24, 25]. First, BCR‐ABL alone or in combination with NUP98‐HOXA9 was retrovirally introduced into hematopoietic stem/progenitor cells (HSPCs) obtained from 5‐fluorouracil pretreated donor mice (Figure S7A,B). These transduced cells were then transplanted into lethally irradiated recipients. Next, we silenced SOX4/SMAD3 with shRNA in leukemia cells derived from these primary mice (Figure S7C), and subsequently transplanted these modified leukemia cells into second‐generation recipient mice.

**FIGURE 7:**
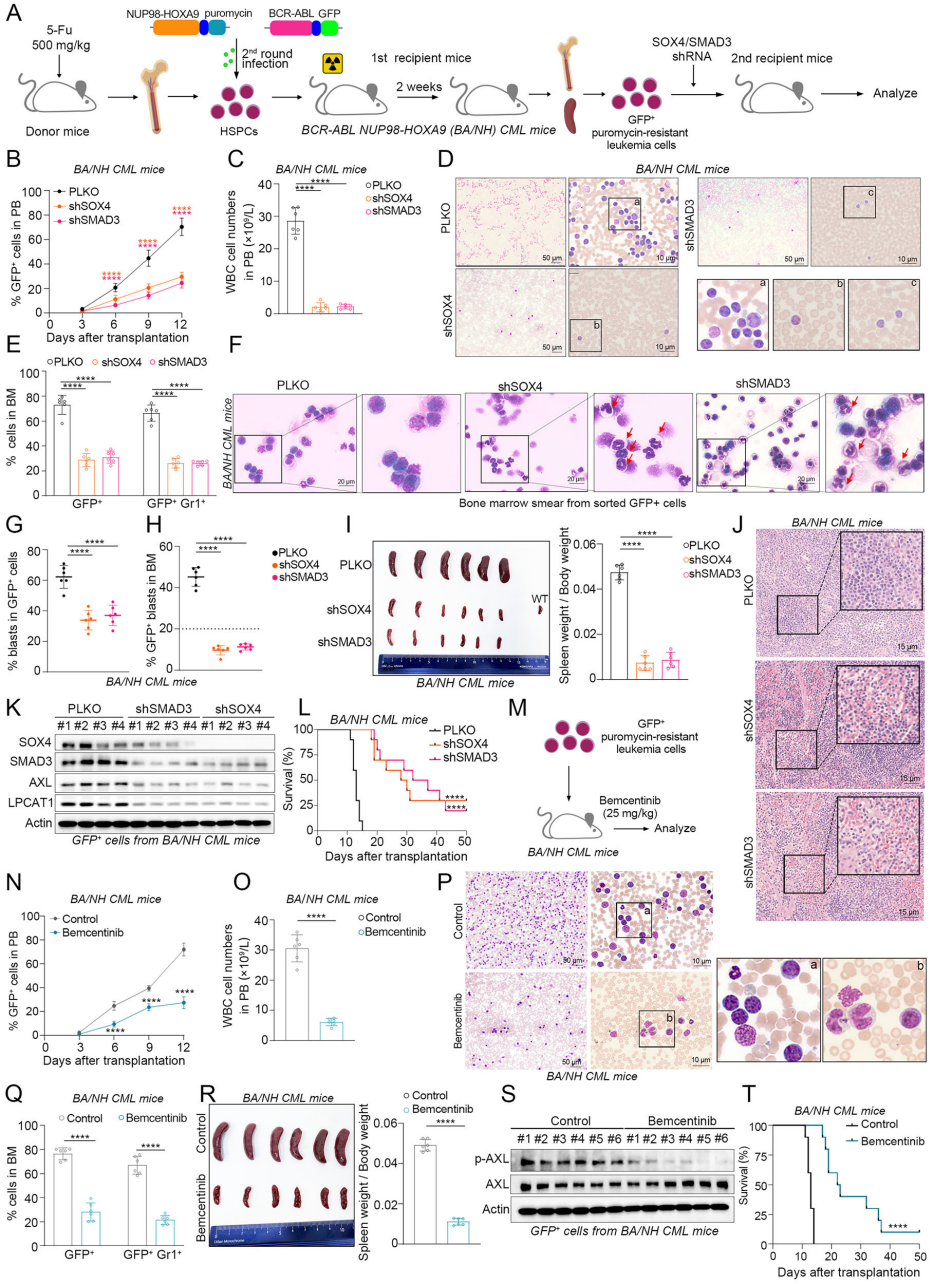
Targeting SOX4/SMAD3‐AXL axis suppresses CML progression in vivo. (A) Schematic of the BCR‐ABL/NUP98‐HOXA9(BA/NH) driven CML mouse model establishment and intervention strategy. (B) Percentages of GFP^+^ cells in PB from BA/NH CML mice of the SOX4/SMAD3 knockdown group. n = 6 mice per group. (C) Total number of WBCs in PB from BA/NH mice transplanted with vector or SOX4/SMAD3 shRNA‐transduced leukemia cells. n = 6 mice per group. (D) Representative Wright‐Giemsa staining of PB smears from BA/NH CML mice transplanted with vector or SOX4/SMAD3 shRNA‐transduced leukemia cells. (E) Percentages of GFP^+^ and GFP^+^Gr1^+^ cells in BM from BA/NH CML mice of the SOX4/SMAD3 knockdown group. n = 6 mice per group. (F) Representative Wright‐Giemsa staining of BM smears from BA/NH CML mice of the SOX4/SMAD3 knockdown group. (G) The proportion of blast cells was analyzed in bone marrow smears prepared from sorted GFP^+^ cells following SOX4/SMAD3 knockdown in BA/NH CML mice. (H) The proportion of GFP^+^ blast cells was analyzed in bone marrow cells from BA/NH CML mice with SOX4/SMAD3 knockdown. (I) Gross pathology (left) and relative weights (right) of the spleens from BA/NH CML mice of the SOX4/SMAD3 knockdown group. n = 6 mice per group. (J) HE staining of the spleens from BA/NH CML mice with SOX4 or SMAD3 knockdown. (K) Western blot analysis of indicated protein expression in leukemia cells from BA/NH CML mice. n = 4 mice per group. (L) Kaplan‐Meier survival curves of BA/NH mice transplanted with vector or SOX4/SMAD3 shRNA‐transduced leukemia cells. n = 10 mice per group. (M) Experimental schema of Bemcentinib treatment in the BA/NH CML mouse model. (N) The percentage of GFP^+^ cells in the PB of BA/NH CML mice treated with Bemcentinib. n = 6 mice per group. (O) Total number of WBCs in the PB of BA/NH CML mice treated with Bemcentinib. n = 6 mice per group. (P) Wright‐Giemsa staining of PB smears of BA/NH CML mice treated with Bemcentinib. n = 6 mice per group. (Q) Percentages of GFP^+^ and GFP^+^Gr1^+^ cells in BM from BA/NH CML mice of the Bemcentinib‐treated group. n = 6 mice per group. (R) Gross pathology (left) and relative weight (right) of the spleens from BA/NH CML mice treated with Bemcentinib. n = 6 mice per group. (S) Western blot analysis of the total and phosphorylation levels of AXL in leukemia cells from BA/NH CML mice. n = 6 mice per group. (T) Kaplan‐Meier survival curves of Bemcentinib‐treated versus vehicle‐treated BA/NH CML mice. n = 10 mice per group. Data are presented as mean ± s.d. Two‐way ANOVA Dunnett's multiple comparisons test were performed in (B‐N), two‐tailed *t*‐tests in (O,Q), log‐rank (Mantel‐Cox) test in (L) and (T). n represents the number of biological replicates. *p < 0.05, **p < 0.01, ***p < 0.001, ****p < 0.0001; ns, not significant. See also Figures S7–S9.

Regardless of the genetic context (BA or BA/NH CML mice), knockdown of either SOX4 or SMAD3 consistently suppressed disease progression (Figure 7A, S7D). The results showed that GFP^+^ leukemia cells and the total white blood cell (WBC) number were reduced in peripheral blood (PB) of SOX4 or SMAD3 knockdown CML mice (Figure 7B–D, S7E–G). Meanwhile, SOX4/SMAD3 deficiency significantly inhibited the engraftment of GFP^+^ myeloid leukemia cells in the bone marrow (BM) of recipient mice (Figure 7E, S7H). This was characterized by a differentiation tendency among GFP^+^ cells (Figure 7F,G, S7I,J), which correlated with a significant decrease in the blast cell percentage in the total BM (Figure 7H, S7K). Moreover, TFs knockdown ameliorated the severity in splenomegaly and attenuated leukemic cell infiltration (Figure 7I,J, S7L,M). Flow cytometry‐sorted GFP^+^ leukemia cells revealed mutual regulation of SOX4 and SMAD3 at the mRNA and protein level in vivo, as well as their regulatory effects on AXL and LPCAT1 (Figure 7K, S7N,O). In parallel, untargeted lipidomics analysis revealed that SOX4/SMAD3 knockdown induced substantial lipid dysregulation in BA CML mice, characterized by significant alterations in phospholipid species, particularly PC (Figure S8A–C). Further analysis of phospholipid saturation demonstrated that, consistent with observations in human CML‐BP cell lines, TFs knockdown reduced both the proportions of SFA‐PL and MFA‐PL (Figure S8D,E). To mechanistically consolidate these findings, we performed CUT&Tag analysis in leukemic cells from BA CML mice, which confirmed that SOX4 and SMAD3 predominantly co‐occupied both promoter and enhancer regions genome‐wide (Figure S8F,G). Importantly, we observed mutual binding of SOX4 and SMAD3 at each other's promoter and enhancer regions (Figure S8H). Furthermore, both transcription factors directly bound to regulatory elements of AXL and LPCAT1(Figure S8H), establishing a reinforced transcriptional axis where SOX4 and SMAD3 autoregulate their expression while coordinately controlling key downstream effectors to drive CML progression. Importantly, knockdown of SOX4 or SMAD3 significantly prolonged the survival of CML‐BP mice, highlighting the functional necessity of this axis in disease maintenance (Figure 7L, S7P).

Having explored the effects of the AXL inhibitor Bemcentinib on CML cells in vitro, we next used the two CML mouse model to examine its in vivo effects (Figure 7M, S9A). As expected, Bemcentinib treatment significantly reduced GFP^+^ leukemia cells in PB (Figure 7N, S9B). Consistent with SOX4/SMAD3 knockdown, Bemcentinib treatment decreased WBC count, alleviated leukemia cells burden in BM, promoted cell differentiation, and alleviated splenic pathological severity (Figure 7O–R, S9C–M). Immunoblotting results showed that Bemcentinib significantly blocked the activation of AXL in leukemia cells (Figure 7S, S9N). In addition, the treatment was well‐tolerated, with no significant changes in body weight (Figure S9O). Meanwhile, serum samples were collected to assess liver and kidney toxicity. The ALT, AST and CR levels of Bemcentinib‐treated mice were comparable to controls (Figure S9P). In summary, pharmacologic inhibition of AXL delayed the progression of CML, and Bemcentinib represents a promising therapeutic strategy for CML (Figure 7T, S9Q).

## 3 Discussion

The transcriptional dysregulation mediated by SEs has been extensively investigated in the occurrence and development of other cancers, but it has rarely been reported in the progression of CML. Here, we performed H3K27ac ChIP‐seq and RNA‐seq on tissue samples from CML patients at different clinical stages. Our experimental design prioritized consistency with public CML datasets also generated from mononuclear cells to ensure direct comparability. This approach also provided the cell numbers required for robust ChIP‐seq by avoiding prohibitive cell loss from further purification. We acknowledge the inherent cellular heterogeneity as a limitation. Future studies will employ advanced single‐cell technologies like scRNA‐seq and scCUT&Tag to decipher transcriptional and chromatin landscapes in homogeneous populations. Following an integrative multi‐omics analysis and rigorous filtering, we identified 4 candidate transcription factors. Interestingly, SOX4 and SMAD3 showed a high correlation only in CML‐BP and we further validated that SOX4 and SMAD3 regulate each other's transcription by co‐binding to each other's and their own enhancer or promoter elements. Cooperative binding of TFs to regulatory regions orchestrates highly cooperative transcriptional activation and is essential for key biological processes [26, 27]. Notably, SOX4 and SMAD3 cooperatively bind with less than 150 bp spacing, mediated by nucleosomes, as well as direct protein‐protein interactions, reinforcing transcriptional complex stability.

SOX4 acts as an oncogene in acute leukemia, sustaining self‐renewal and impeding differentiation in C/EBPα‐mutant AML [28], and enabling oncogenic survival signals in BCR‐ABL‐driven B‐ALL [29]. Consistently, SOX4 expression was downregulated in progression‐free CML patients following imatinib treatment [30]. SMAD3, a TGF‐β signaling effector, cooperates with FOXO3a in regulating transcription and is required for CML stem cell maintenance [31]. However, the roles of SOX4 and SMAD3 in CML transformation have not been previously reported.

In our study, we found that SOX4 and SMAD3 synergistically accelerate the malignant progression of CML in vitro and in vivo. To elucidate the mechanisms, we performed RNA‐seq pathway enrichment analysis after knockdown of each TF. We prioritized two overlapping high‐ranking pathways (Signaling by Receptor Tyrosine Kinases and lipid metabolism) for further investigation due to their relevance in CML biology. Increased activation of several tyrosine kinases other than BCR‐ABL in imatinib‐ and nilotinib‐resistant CML cell lines and patients [32]. Therefore, targeting non‐BCR‐ABL kinases may be a potential option to treat CML patients. Among the candidate genes in the “Signaling by Receptor Tyrosine Kinases” pathway, we found that SOX4 and SMAD3 directly regulate AXL by binding to its promoter and enhancer. Our investigations uncover SOX4/SMAD3 regulate the expression of AXL, thereby activating its downstream oncogenic signaling pathways, ultimately promoting the progression of CML. Notably, the AXL inhibitor Bemcentinib markedly reduced proliferation and progression of CML cells in vitro and in vivo. Moreover, studies have shown the metformin targets SOX4 degradation in various tumors [33], and we will explore its potential combination with conventional CML therapy.

The signaling pathways mediated by receptor tyrosine kinases is regulated by colocalization of the ligand, receptor, and its downstream effectors into lipid raft/caveolin‐1‐enriched membrane microdomains [34]. The membrane receptor AXL functions dependently by binding its ligand GAS6 [35]. Bone marrow‐derived stromal cells‐secreted GAS6 activates the AXL on leukemia cells to form a paracrine regulatory loop [36]. Thus, the membrane localization of AXL is critical for ligand binding and signaling pathway activation. Surprisingly, we found that SOX4/SMAD3 knockdown not only reduced AXL expression, but also reduced its membrane localization. Membrane receptor localization and signaling activity are influenced by the biophysical properties of the cell membrane, including curvature, charge, order, fluidity, and architecture [37]. As suggested by the pathway enrichment, SOX4/SMAD3 regulate lipid metabolism, specifically PC metabolism. PC is synthesized de novo through the Kennedy process and then undergo deacylationreacylation remodeling via the Lands’ cycle [38]. The Lands’ cycle efficiently diversifies membrane phospholipid by modifying fatty acid saturation and other biochemical features [39]. The reacylation phase of the Lands’ cycle is catalyzed by lysophosphatidylcholine acyltransferases, LPCATs. We confirmed that SOX4/SMAD3 can directly regulate the key enzyme of phospholipid metabolism, LPCAT1, thereby altering the saturated phospholipid content in the membrane, and reshaping the membrane structure, order and fluidity. Exogenous overexpression of LPCAT1 or supplementation with its metabolite DPPC, a saturated PC [40], reversed the internalization of AXL and restored its membrane localization. It was previously reported that LPCAT1‐mediated membrane lipid remodeling can promote tumor progression in various ways, such as promote ferroptosis evasion [41] and sustain oncogenic activity of EGFR [42]. However, SOX4 and SMAD3 may also regulate membrane phospholipid remodeling pathways beyond LPCAT1, such as regulating SLC44A2, which affects choline transport [43], regulating STARD10 and PCTP, which affect PC transport between endoplasmic reticulum and plasma membrane [44, 45]. These limitations are what we need to break through and solve in the future.

## 4 Conclusion

In this study, we identified a specific transcriptional regulatory network driven by the synergy of SOX4/SMAD3, specifically SOX4 and SMAD3 not only directly regulate the expression of AXL, but also affect its membrane localization and activation by regulating LPCAT1‐mediated membrane phospholipid remodeling, both of which play important roles in the progression of CML. These findings enhance our understanding of transcriptional dysregulation in CML transformation, and provide potential therapeutic strategies.

## 5 Materials and methods

The cell lines, patient samples, antibodies, reagents and sequence information (including RRID) involved in this study are all described in detail in the Supplementary table.

### 5.1 Cell lines

LAMA‐84, K562, KU812, HEK293T cells were purchased from American Type Culture Collection (ATCC). Ba/F3 BCR/ABL‐T315I cell lines were kindly provided as a gift by Dr. BZ. Carter at the University of Texas M. D. Anderson Cancer Center (Houston, USA). All the above cell lines have been confirmed by STR identification to be free from contamination. The cell lines K562, LAMA‐84, KU812, KBM5 were all derived from CML‐BP patients. KBM5 cells, which express the wild‐type BCR‐ABL, were derived from a female CML patient. The KBM5‐T315I subline was originally established by exposure to increasing concentrations of STI571. KBM5‐T315I cells harbor a threonine‐to‐isoleucine substitution at position 315 of ABL, conferring resistance to Imatinib. The Ba/F3 BCR/ABL‐T315I cell line is a murine Ba/F3 cell line generated by plasmid transfection to express the BCR/ABL T315I. The cell culture conditions are shown in Supplementary Table S3.

### 5.2 Patient Cohort and Ethics Statement

The study was approved by the ethics committee of the Seventh Affiliated Hospital of Sun Yat‐sen University. All samples were obtained from the Seventh Affiliated Hospital of Sun Yat‐sen University with prior written informed consent from all participants. This study utilized primary samples from a total of 40 CML patients (19 in chronic phase, CML‐CP, and 21 in blast phase, CML‐BP). The CML‐BP group contained 10 females and 11 males, with a median age of 41 years (range 28–70). The CML‐CP group consisted of 7 females and 12 males, with a median age of 30 years (range 18–81). The diagnosis of CML‐CP and CML‐BP was strictly defined according to the WHO classification and confirmed through a combination of clinical presentation, peripheral blood counts, bone marrow morphology, cytogenetics, and molecular testing1. The key criterion was the blast percentage, with CML‐BP defined by ≥20% blasts and CML‐CP by <10% blasts in the peripheral blood or bone marrow. The clinical and biological characteristics of the 40 patients were shown in Supplementary Table S4.

### 5.3 Isolation of Human Mononuclear Cells

Patients peripheral blood or bone aspiration was collected in EDTA tubes, and mononuclear cells were then isolated by Ficoll density gradient centrifugation. The cells were aliquoted and stored at −80°C for subsequent RNA‐seq and ChIP‐seq library preparation, respectively.

### 5.4 Chromatin Immunoprecipitation and CUT&Tag

ChIP was performed using our previously‐described methods [9]. In brief, 1 × 10^7^ mononuclear cells from patients were fixed with 1% paraformaldehyde for 10 min at room temperature, followed by quenching with an equal volume of 250 mM glycine. After washing with PBS, cells were lysed in ice‐cold lysis buffer for 30 min. The pellets were then collected by centrifugation, resuspended in shearing buffer, and sonicated to fragment genomic DNA into 300–500 bp fragments. For immunoprecipitation, solubilized chromatin was incubated with target‐specific or control IgG antibodies overnight at 4°C with rotation. Antibody‐chromatin complexes were captured using magnetic beads, and bound DNA was eluted with elution buffer. Following reverse cross‐linking and RNA digestion, immunoprecipitated DNA was purified with the MinElute PCR Purification Kit (Qiagen) and subsequently analyzed by qPCR or used for library preparation and sequencing on the Illumina HiSeq 4000 platform. The primers used for ChIP‐qPCR are provided in Supplementary Table S5.

CUT&Tag was performed following the manufacturer's instructions (Vazyme). Briefly, 1 × 10^5^ cells were harvested and lysed with NE buffer for 10 min on ice. Samples were then centrifuged and resuspended in wash buffer. Nuclei were bound to ConA magnetic beads for 10 min at room temperature. Then conA‐bound nuclei were suspended and incubated with specific primary antibodies in 50 µL antibody binding buffer overnight at 4°C. After incubation, Samples were washed to remove unbound primary antibody, resuspended in Dig‐wash buffer containing the secondary antibody and incubated for 1 h at room temperature. Samples were subsequently washed in Dig‐300 buffer and resuspended in tagmentation buffer (Dig‐300 buffer plus TTBL), followed by incubation at 37°C for 1 h to complete the Tn5 tagmentation reaction. Samples were then incubated in a thermocycler with a heated lid at 55°C for 10 min to release Tn5 and prepare tagmented chromatin for PCR. Purified fragmented DNA was mixed with 5 µL of barcoded i5 primer, 5 µL of barcoded i7 primer and 25 µl of 2× CAM PCR mix. Samples were then placed in a thermocycler and PCR amplification was performed using 9∼13 rapid cycles. CUT&Tag libraries were purified using VATHS DNA Clean Beads (Vazyme) at a 2:1 (vol/vol) ratio of beads to sample, quantified on a TapeStation Bioanalyzer instrument (Thermo Fisher) and pooled for sequencing. The information of the antibodies used is shown in Supplementary Table S6.

### 5.5 Super Enhancer Analysis

Super‐enhancers were identified using the ROSE algorithm, which merges enhancer regions within a 12 kb stitching distance and ranks the merged regions based on their signal intensity. SEs were defined as regions with a signal intensity above the inflection point cutoff (>1). Based on this, we integrated SE data from CML‐BP and CML‐CP samples and performed a differential binding analysis using DiffBind (version 3.4.11). Subsequently, the genomic distance between each SE and all gene transcription start sites (TSSs) was calculated, and SEs were assigned to their closet target gene using the ROSE_geneMapper.py tool with default parameters. Finally, differential SEs were filtered by applying thresholds of |log2(fold change)| > 0.584 and FDR < 0.05.

### 5.6 Total RNA Extraction

Total RNA was isolated from both patient‐derived samples and cell lines using the RNeasy Mini Kit (Qiagen) according to the manufacturer's instructions. In brief, cells were lysed using the provided RLT lysis buffer supplemented with β‐mercaptoethanol to ensure complete disruption and RNA stabilization. The lysate was then mixed with ethanol and applied to the RNeasy silica membrane. Contaminants were effectively removed through sequential washes with RW1 buffer and RPE buffer. Following the wash steps, RNA was eluted in RNase‐free water. RNA concentration and purity were determined spectrophotometrically using a NanoDrop instrument.

### 5.7 RNA‐Seq Analysis

Total RNA was extracted and then submitted to BGI (Wuhan, China) for commercial library construction and RNA sequencing services on the Illumina platform. Raw reads obtained from high‐throughput sequencing were quantified at the transcript level using Salmon (version 0.13.1) with the hg38 (GENCODE v44) transcriptome as the reference. Transcript‐level abundances (TPM) were then summarized to gene levels using the tximport Bioconductor package (version 1.27.1). Differentially expressed genes (DEGs) were identified using the DESeq2 package (version 1.34.0) with adjusted *p*‐value < 0.05 and absolute log 2 (fold change) >0.584. Low‐expression genes (average abundance≤1) were filtered out to ensure robust results. Pathway enrichment analysis was conducted to explore the biological significance of DEGs, and volcano plots were generated to visualize the distribution of significantly differentially expressed genes.

### 5.8 Principal Component Analysis

Raw reads were aligned to the hg19 reference genome using HISAT2. Subsequently, read counts were obtained using HTSeq, and normalized to gene expression levels using RPKM (Reads Per Kilobase per Million mapped reads). Based on the RPKM‐normalized data, the top 4500 most variable genes were selected by calculating the standard deviation (SD) of gene expression across samples. Principal Component Analysis was then performed using the specified function in the FactoMineR R package (version 2.8), and the first two principal components (PC1 and PC2) were visualized using the fviz_pca_ind function to explore sample relationships and group separation.

### 5.9 Lipidomic analyses

The experiments and data analysis were supported by Shanghai Applied Protein Technology Co., Ltd. For sample preparation and lipid extraction, lipids were extracted according to methyl tert‐butyl ether (MTBE) method (Sigma Aldrich). Briefly, samples were first spiked with appropriate amounts of internal lipid standards and then homogenized with 200 µL ice water and 240 µL methanol (pre‐cooled overnight at −80°C). Subsequently, 800 µL of MTBE was added, and the mixture was ultrasonicated for 20 min at 4°C followed by sitting still for 30 min at room temperature. The solution was centrifuged at 15 000 × g for 5 min at room temperature and the supernatant was collected and dried under nitrogen.

For LC‐MS/MS lipid analysis, reverse‐phase chromatography was selected for LC separation using 15 cm Accucore Vanquish C18 column (1.5 µm particle size, 2.1 mm diameter). The lipid extracts were resuspended in 200 µL of 90% isopropanol/acetonitrile, centrifuged at 14 000 × g for 15 min, and finally 3 µL of sample was injected. Solvent A consisted of acetonitrile–water (6:4, vol/vol) with 0.1% formic acid and 0.1 mM ammonium formate, and solvent B consisted of acetonitrile–isopropanol (1:9, vol/vol) with 0.1% formic acid and 0.1 mM ammonium formate. The initial mobile phase was 30% solvent B at a flow rate of 300 µL/min, held for 2 min, then linearly increased to 100% solvent B in 23 min, followed by equilibrating at 5% solvent B for 10 min. Mass spectra were acquired by Q‐Exactive (Thermo Fisher) system in positive and negative modes. The data acquired from the above lipidomics method were imported into LipidSearchTM software (Thermo Fisher) for lipid identification and normalized quantification analysis.

### 5.10 Chromosome Conformation Capture Assay

The 3C assay was performed following established literature protocols [46] and manufacturer's instructions (BersinBio, Cat# Bes5006). Briefly, 2 × 10^7^ LAMA‐84 cells were crosslinked with 1% formaldehyde for 10 min at room temperature with rotation. The reaction was quenched with 1.375 M glycine, followed by two washes with cold PBS. Cells were lysed in ice‐cold lysis buffer (10 mM Tris‐HCl, pH 8.0; 10 mM NaCl; 0.2% NP‐40) supplemented with protease inhibitors for 15 min on ice. After 20 rounds of bouncing, nuclei were pelleted and resuspended in 1× restriction enzyme buffer. Chromatin was digested with HindIII (200 U) at 37°C for 6 h with rotation, followed by heat inactivation. Ligation was carried out using 700 U of T4 DNA ligase at 16°C for 2 h. After proteinase K treatment to reverse crosslinks and RNase A digestion, DNA was purified through phenol‐chloroform and analyzed by qPCR using primers listed in Supplementary Table S5. The final PCR products were subjected to Sanger sequencing.

### 5.11 ChIP‐re‐ChIP Assay

The ChIP‐Re‐ChIP assay was conducted following established methodologies [47, 48]. Briefly, 1 × 10^8^ LAMA‐84 cells were crosslinked with 1% formaldehyde and chromatin was fragmented by sonication. The cell lysates were incubated overnight at 4°C with primary antibodies against SOX4 or SMAD3. After incubation with ChIP‐grade Protein G Magnetic Beads, the beads were washed and bound complexes were eluted using elution buffer at 37°C for 30 min. The eluates then underwent a second round of immunoprecipitation with the corresponding secondary antibody or control IgG. Following Re‐ChIP, immunocomplexes were eluted and reverse crosslinking was performed. DNA was purified using the MinElute PCR Purification Kit (Qiagen), quantified by qPCR, and normalized to input DNA. All primers used for ChIP‐qPCR are provided in Supplementary Table S5.

### 5.12 Generation of Lentivirus and Retrovirus

The shRNAs, sgRNAs, or DNA expression constructs were co‐transfected into HEK293T cells with the pSPAX2 and pMD2.G packaging constructs. The supernatants containing viral particles were collected after transfection for 48 h or 72 h. The sequences of the shRNAs and sgRNAs are provided in Supplementary Table S7.

The BCR‐ABL retroviruses were generated by transient transfection with the MIRG1‐BCR‐ABL‐GFP and Ecopack constructs in HEK293T cells and harvested after transfection for 48 or 72 h.

### 5.13 Purification of Murine HSPCs Cells From Bone Marrow

The purification of murine hematopoietic stem/progenitor cells (HSPCs) was performed according to an established methodology [49]. Briefly, 5‐week‐old female BALB/c or C57BL/6 mice were intravenously administered 5‐fluorouracil (5‐FU; 200 mg/kg) 5 days in advance to stimulate HSPC mobilization. Bone marrow cells were then harvested from femurs and tibias, and mononuclear cells were isolated using Ficoll‐Paque density gradient centrifugation. The cells were stained with a biotin‐conjugated lineage antibody cocktail (Anti‐CD5, CD45R, CD11b, Gr‐1, and Ter‐119), and lineage‐positive cells were depleted with anti‐biotin MicroBeads. The resulting lineage‐negative cells were further stained with APC‐conjugated anti‐c‐kit and Alexa Fluor 700‐conjugated anti‐Sca‐1 antibodies for the identification and isolation of the Lin^−^ Sca‐1^+^ c‐kit^+^ (LSK) population.

### 5.14 Cell Viability Assay

Cells were seeded into 96‐well plates at a density of 1 × 10^4^ cells per well and treated with rising concentrations of Bemcentinib for 48 h. Subsequently, 10 µL of CCK‐8 reagent was added to each well. After 3 h of incubation, Absorbance was detected at wavelength of 450 nm using a 96‐well plate reader.

### 5.15 Soft‐Agar Colony Formation Assay

A predetermined number of CML cells were suspended in 0.3% low‐gelling‐temperature agaros with 20% (vol/vol) FBS, gently mixed and then plated onto 12‐well plates. Each shRNA condition was performed in triplicate. After agarose solidification, the plates were cultured in 37°C, 5% CO_2_ incubator. Cells were replenished with fresh growth medium every 3 days. Colonies were quantified and imaged for subsequent analysis after 2 weeks.

### 5.16 Immunoblotting Assay

Cells were lysed in RIPA lysis buffer on ice for 30 min, and the protein concentrations were determined by BCA assay. After quantification, proteins were separated by SDS‐PAGE and transferred to PVDF membrane (Millipore). The membranes were blocked with 5% milk for 60 min at room temperature, and then incubated with specific primary antibodies overnight at 4°C. After washing with PBST, the membranes were incubated with secondary antibodies for 60 min at room temperature. Following another PBST wash, immunoreactive bands were visualized using chemiluminescence. Indicated antibodies are listed in Supplementary Table S6.

### 5.17 Immunofluorescence

CML cells were first adhered to polylysine‐coated glass slides, and fixed with 4% paraformaldehyde (Thermo, 28908) for 10 min. After fixation, the cells were washed and blocked with 1% BSA for 30 min, followed by incubation with specific primary antibody overnight at 4°C. Afterward, the cells were incubated with an appropriate second antibody at room temperature for 60 min. Finally, the nuclei were stained with DAPI. Zeiss Laser Scanning Microscopy (LSM) 880 with is used to perform the imaging acquisition. Indicated antibodies are listed in Supplementary Table S6.

### 5.18 Flow Cytometry and Cell Sorting

For flow cytometric analyses of cell surface differentiation markers, 3 × 10^5^ cells were collected, blocked with 1% BSA for 30 min at 4°C, and then incubated with specific antibody for 60 min at 4°C. After incubation, the cells were washed twice with cold PBS for flow cytometric analysis. For cell cycle and cell apoptosis analyses, cells were prepared following product instructions (Sungene Biotech) and then analyzed by flow cytometry. Flow cytometry was performed on Beckman flow cytometry and data were analyzed with FlowJo software (Treestar). Fluorescence activated cell sorting (FACS) of GFP^+^ leukemia cells was performed by BD Biosciences system, following the manufacturer's instructions. Indicated antibodies are listed in Supplementary Table S3: Antibodies.

### 5.19 Luciferase Reporter Assay

Candidate DNA regions (500∼1000 bp) were amplified by PCR and subsequently cloned into either the pGL3‐Promoter luciferase reporter vector or the pGL3‐Basic luciferase reporter vector (Promega). The constructed plasmids were verified by Sanger sequencing. LAMA‐84 cells were transfected using electroporation (NEPA GENE). For normalization purposes, the Renilla luciferase control vector was co‐transfected. Luciferase activity was measured 48 h post‐transfection using the Dual‐Luciferase Reporter Assay System (Promega).

### 5.20 RT‐qPCR

The purified 1 µg RNA for each sample was reverse‐transcribed into complementary DNA (cDNA) using the Strand cDNA Synthesis SuperMix (Yeasen). Quantitative real‐time PCR was performed on the CFX384 Real‐Time PCR Detection System (Bio‐Rad) using gene‐specific primers and SYBR Green Master Mix (Yeasen) by following the manufacturer's instructions. GAPDH served as an endogenous control for normalization, and the relative mRNA expression levels were calculated using the 2−ΔΔCT method. The primer sequences used in this study are listed in Supplementary Table S6.

### 5.21 CML Model and Bemcentinib Therapy

All animal procedures were conducted in accordance with the ethical guidelines and were approved by the Institutional Animal Care and Use Committee of Guangzhou Medical University (Approval No. GY2024‐772). The study strictly adhered to all relevant ethical regulations governing animal research, with predefined humane endpoints including >15% body weight loss, impaired mobility, or other signs of distress requiring euthanasia.

The CML model was conducted following established methodologies [23, 24, 25, 50]. Murine HSPCs were harvested from 5‐week‐old donor female mice matching the recipient strain for each model. These HSPCs were then subjected to two rounds of retroviral transduction via spinfection (1500 × g, 90 min, 32°C) in medium supplemented with interleukin‐3 (IL‐3, 6 ng/mL), interleukin‐6 (IL‐6, 6 ng/mL), and stem cell factor (SCF, 50 ng/mL). Viruses used were as follows: MIRG1‐BCR‐ABL‐GFP was used to generate the single‐transduction model (BA CML mice), while MIRG1‐BCR‐ABL‐GFP together with MIRG1‐NUP98‐HOXA9‐puromycin were used for the co‐transduction model (BA/NH CML mice). Subsequently, 1 × 10^6^ transduced cells were transplanted into sub lethally irradiated (4.5 Gy) 5‐week‐old female recipient mice to generate primary models. Specifically, BA‐transduced HSPCs (derived from BALB/c donors) were transplanted into BALB/c recipients, whereas BA/NH‐*co*‐transduced HSPCs (derived from C57BL/6 donors) were transplanted into C57BL/6 recipients. Engraftment was typically observed approximately 2 weeks later.

To evaluate the in vivo effect of SOX4 or SMAD3 knockdown, leukemia cells isolated from the primary CML mice were transduced twice with PLKO.1, murine SOX4‐, or SMAD3 ‐specific shRNA lentiviruses (1500 × g, 90 min, 32°C. The transduced cells (1 × 10^6^ per mouse) were then transplanted into 5‐week‐old female recipient mice. Mice were monitored daily for clinical signs and physiological parameters to track CML progression. For Bemcentinib treatment, mice received the drug (25 mg/kg) via oral gavage twice daily, starting on the second day after transplantation.

### 5.22 Phospholipid Liposomes Preparation and Lipid Treatment

The phospholipids DPPC(PC16:0/16:0) (1,2‐dipalmitoyl‐sn‐glycero‐3‐phosphocholine) and POPC (PC16:0/18:1) (1‐palmitoyl‐2‐oleoyl‐sn‐glycero‐3‐phosphocholine) (Avanti Polar Lipids) in chloroform were initially evaporated under a nitrogen stream, followed by completely drying under high vacuum. The thin lipid film was suspended in HBS buffer and incubated in 55°C water bath for 20 min with occasional vortexing to ensure proper hydration. The lipid suspensions were then loaded into a MiniExtruder system (Avanti Polar Lipids) and passed through a 0.1 mm pore size polycarbonate membrane to generate 1 mmol/L liposomes stocks. 10 µM of PC liposomes and an equal amount of HBS buffer were used to treat cells.

### 5.23 Fluorescence Recovery After Photobleaching Analysis

CML cells were seeded overnight on confocal dishes coated with fibronectin. The cells were stained with CellMask Green cell stain (Thermo, Cat# C37608) at 37°C in the dark for 10 min and washed with fresh medium three times. The Zeiss Laser Scanning Microscope (LSM) 900 is used to perform imaging acquisition and FRAP. All live‐cell image was conducted at 37°C, 5% CO_2_ in medium supplemented with 10% (vol/vol) FBS, with each sample imaged for no more than 90 min. For FRAP imaging, the ZEN lite software was used to control image acquisition and exposure. Three regions were selected for FRAP analysis: (1) the bleaching region, (2) the total cell region, and (3) the background region. Photobleaching was achieved by focusing 488 laser on the designated membrane region and continuously exposing it until the fluorescence intensity reached the set threshold. Fluorescent images and fluorescence intensity values of designated region were recorded before and after photobleaching in time‐series scan mode.

### 5.24 Statistical analysis

All statistical analyses and graphs were performed using GraphPad Prism 8.0 software. Two‐tailed unpaired Student's t‐test was used for comparing two experimental groups, and one‐way or two‐way analysis of variance (ANOVA) was used for comparing three or more experimental groups, and chi‐square test was used for statistics of differences in count data between groups. The Shapiro‐Wilk normality test was employed to verify that the data met the assumptions. Statistical significance was defined as follows: *p* < 0.05 (*), *p* < 0.01 (**), *p* < 0.001 (***) and *p* < 0.0001 (****); ns, not significant.

## Author Contributions

E.Z.L., L.L.J., Z.G.L., P.L.L., Y.Y.Z., and X.P.S. designed the research. E.Z.L., L.L.J., X.Y.L. L.D.M., H.C.Z., J.X.F., B.L., Y.M., J.H.C., A.C.L., W.H.L., Y.Y.G., and Z.R.G. performed the experiments and analyzed and interpreted the data. G.J.P., Q.M., L.Z.J., S.W.H., A.D.H., and X.L.Y. analyzed and interpreted the data. E.Z.L. and X.P.S. wrote the manuscript. X.P.S. supervised the entire study. All authors reviewed the manuscript

## Funding

This work was supported by the National Natural Science Foundation of China (82170177/H0809) to XPS; Shenzhen Medical Research Fund (B2402019) to XPS and YYZ; Natural Science Foundation of Guangdong Province (2023A1515011976) and the Innovation team of general Universities in Guangdong Province (2022KCXTD021) to XPS; National Natural Science Foundation of China (82303536) and the Natural Science Foundation of Guangdong Province (2025A1515010451) to LLJ; Guangdong Basic and Applied Basic Research Foundation (2024A1515010703) and National Natural Science Foundation of China (32200538) to YYZ. National Natural Science Foundation of China (82270161) and Guangdong Basic and Applied Basic Research Foundation (2024B1515020054) to PLL.

## Conflicts of Interest

The authors declare no conflicts of interest.

## Supporting information




**Supporting File 1**: advs74452‐sup‐0001‐SuppMat.docx.

**Supporting File 2**: advs74452‐sup‐0002‐Supplementary Table.xlsx.

## Data Availability

The complete RNA‐seq, ChIP‐Seq, CUT&Tag datasets are available in the Gene Expression Omnibus under accession numbers GSE292716, GSE292717, GSE292718 and in the ArrayExpress database under E‐MTAB‐15893. The public data analyzed in this project include EGAS00001003071.

## References

[1] E. Jabbour and H. Kantarjian , “Chronic Myeloid Leukemia: 2025 Update on Diagnosis, Therapy, and Monitoring,” American Journal of Hematology 99 (2024): 2191–2212, 10.1002/ajh.27443. 39093014

[2] E. Jabbour and H. Kantarjian , “Chronic Myeloid Leukemia,” Jama 333 (2025): 1618–1629, 10.1001/jama.2025.0220. 40094679

[3] D. Perrotti , C. Jamieson , J. Goldman , and T. Skorski , “Chronic Myeloid Leukemia: Mechanisms of Blastic Transformation,” Journal of Clinical Investigation 120 (2010): 2254–2264, 10.1172/JCI41246. 20592475 PMC2898591

[4] J. Senapati , E. Jabbour , H. Kantarjian , and N. J. Short , “Pathogenesis and Management of Accelerated and Blast Phases of Chronic Myeloid Leukemia,” Leukemia 37 (2023): 5–17, 10.1038/s41375-022-01736-5. 36309558

[5] J. E. Bradner , D. Hnisz , and A. Y. Richard , “Massachusetts Institute of Technology. Department of Biology,” Cell 168 (2017): 629–643.28187285

[6] I. Sur and J. Taipale , “The Role of Enhancers in Cancer,” Nature Reviews Cancer 16 (2016): 483–493, 10.1038/nrc.2016.62. 27364481

[7] M. Wang , Q. Chen , S. Wang , et al., “Super‐Enhancers Complexes Zoom in Transcription in Cancer,” Journal of Experimental & Clinical Cancer Research 42 (2023): 183, 10.1186/s13046-023-02763-5. 37501079 PMC10375641

[8] S. Heinz , C. E. Romanoski , C. Benner , and C. K. Glass , “The Selection and Function of Cell Type‐Specific Enhancers,” Nature Reviews Molecular Cell Biology 16 (2015): 144–154, 10.1038/nrm3949. 25650801 PMC4517609

[9] X. Shi , Y. Zheng , L. Jiang , et al., “EWS‐FLI1 Regulates and Cooperates With Core Regulatory Circuitry in Ewing Sarcoma,” Nucleic Acids Research 48 (2020): 11434–11451, 10.1093/nar/gkaa901. 33080033 PMC7672457

[10] M. Gartlgruber , A. K. Sharma , A. Quintero , et al., “Super Enhancers Define Regulatory Subtypes and Cell Identity in Neuroblastoma,” Nature Cancer 2 (2021): 114–128, 10.1038/s43018-020-00145-w. 35121888

[11] M. Noura , H. Matsuo , T. Yasuda , S. Tsuzuki , H. Kiyoi , and F. Hayakawa , “Suppression of Super‐Enhancer‐Driven TAL1 Expression by KLF4 in T‐Cell Acute Lymphoblastic Leukemia,” Oncogene 43 (2024): 447–456, 10.1038/s41388-023-02913-1. 38102337

[12] J. Yu , Z. Zhang , Y. Chen , et al., “Super‐Enhancer‐Driven IRF2BP2 Is Activated by Master Transcription Factors and Sustains T‐ALL Cell Growth and Survival,” Advanced Science 12 (2025): 2407113, 10.1002/advs.202407113. 39454110 PMC11714186

[13] S. Kiehlmeier , M.‐R. Rafiee , A. Bakr , et al., “Identification of Therapeutic Targets of the Hijacked Super‐Enhancer Complex in EVI1‐Rearranged Leukemia,” Leukemia 35 (2021): 3127–3138, 10.1038/s41375-021-01235-z. 33911178 PMC8550965

[14] L. S. Qi , M. H. Larson , L. A. Gilbert , et al., “Repurposing CRISPR as an RNA‐guided Platform for Sequence‐Specific Control of Gene Expression,” Cell 152 (2013): 1173–1183, 10.1016/j.cell.2013.02.022. 23452860 PMC3664290

[15] P. Gamas , S. Marchetti , A. Puissant , et al., “Inhibition of Imatinib‐mediated Apoptosis by the Caspase‐Cleaved Form of the Tyrosine Kinase Lyn in Chronic Myelogenous Leukemia Cells,” Leukemia 23 (2009): 1500–1506, 10.1038/leu.2009.60. 19340007

[16] S. Grosso , A. Puissant , M. Dufies , et al., “Gene Expression Profiling of Imatinib and PD166326‐resistant CML Cell Lines Identifies Fyn as a Gene Associated with Resistance to BCR‐ABL Inhibitors,” Molecular Cancer Therapeutics 8 (2009): 1924–1933, 10.1158/1535-7163.MCT-09-0168. 19567819

[17] D. K. Graham , D. DeRyckere , K. D. Davies , and H. S. Earp , “The TAM family: Phosphatidylserine‐Sensing Receptor Tyrosine Kinases Gone Awry in Cancer,” Nature Reviews Cancer 14 (2014): 769–785, 10.1038/nrc3847. 25568918

[18] I. Ben‐Batalla , R. Erdmann , H. Jørgensen , et al., “Axl Blockade by BGB324 Inhibits BCR‐ABL Tyrosine Kinase Inhibitor–Sensitive and ‐Resistant Chronic Myeloid Leukemia,” Clinical Cancer Research 23 (2017): 2289–2300, 10.1158/1078-0432.CCR-16-1930. 27856601

[19] A. Neubauer , A. Fiebeler , D. Graham , et al., “Expression of Axl, a Transforming Receptor Tyrosine Kinase, in Normal and Malignant Hematopoiesis,” Blood 84 (1994): 1931–1941, 10.1182/blood.V84.6.1931.1931. 7521695

[20] H. Li , Z. Liu , L. Liu , et al., “AXL Targeting Restores PD‐1 Blockade Sensitivity of STK11/LKB1 Mutant NSCLC through Expansion of TCF1+ CD8 T Cells,” Cell Reports Medicine 3 (2022): 100554, 10.1016/j.xcrm.2022.100554. 35492873 PMC9040166

[21] T. Sasaki , P. G. Knyazev , N. J. Clout , et al., “Structural Basis for Gas6–Axl Signalling,” The EMBO Journal 25 (2006): 80–87, 10.1038/sj.emboj.7600912. 16362042 PMC1356355

[22] M. F. Renne and R. Ernst , “Membrane Homeostasis beyond Fluidity: Control of Membrane Compressibility,” Trends in Biochemical Sciences 48 (2023): 963–977, 10.1016/j.tibs.2023.08.004. 37652754 PMC10580326

[23] C. Sun , X. Xu , Z. Chen , et al., “Selective Translational Control by PABPC1 Phase Separation Regulates Blast Crisis and Therapy Resistance in Chronic Myeloid Leukaemia,” Nature Cell Biology 27 (2025): 683–695, 10.1038/s41556-024-01607-4. 40102686

[24] Y. Gu , W. Zheng , J. Zhang , et al., “Aberrant Activation of CaMKIIγ Accelerates Chronic Myeloid Leukemia Blast Crisis,” Leukemia 30 (2016): 1282–1289, 10.1038/leu.2016.53. 27012864

[25] A. B. Dash , I. R. Williams , J. L. Kutok , et al., “A Murine Model of CML Blast Crisis Induced by Cooperation between BCR/ABL and NUP98/HOXA9,” Proceedings of the National Academy of Sciences U S A 99 (2002): 7622–7627, 10.1073/pnas.102583199. PMC124303 12032333

[26] L. A. Mirny , “Nucleosome‐Mediated Cooperativity Between Transcription Factors,” Proceedings of the National Academy of Sciences 107 (2010): 22534–22539, 10.1073/pnas.0913805107. PMC3012490 21149679

[27] S. Kim and J. Wysocka , “Deciphering the Multi‐Scale, Quantitative Cis‐Regulatory Code,” Molecular Cell 83 (2023): 373–392, 10.1016/j.molcel.2022.12.032. 36693380 PMC9898153

[28] H. Zhang , M. Alberich‐Jorda , G. Amabile , et al., “Sox4 Is a Key Oncogenic Target in C/EBPα Mutant Acute Myeloid Leukemia,” Cancer Cell 24 (2013): 575–588, 10.1016/j.ccr.2013.09.018. 24183681 PMC4038627

[29] P. Ramezani‐Rad , H. Geng , C. Hurtz , et al., “SOX4 Enables Oncogenic Survival Signals in Acute Lymphoblastic Leukemia,” Blood 121 (2013): 148–155, 10.1182/blood-2012-05-428938. 23152540 PMC3538327

[30] F. Dong , G. Zhang , X. Zhang , X. Liu , N. Wang , and C. Sun , “Aberrantly Expressed Transcription Factors C/EBP and SOX4 Have Positive Effects in the Development of Chronic Myeloid Leukemia,” Molecular Medicine Reports 16 (2017): 7131–7137, 10.3892/mmr.2017.7486. 28901467

[31] K. Naka , Y. Jomen , K. Ishihara , et al., “Dipeptide Species Regulate p38MAPK–Smad3 Signalling to Maintain Chronic Myelogenous Leukaemia Stem Cells,” Nature Communications 6 (2015): 8039, 10.1038/ncomms9039. PMC4560789 26289811

[32] R. Gioia , C. Leroy , C. Drullion , et al., “Quantitative Phosphoproteomics Revealed Interplay Between Syk and Lyn in the Resistance to Nilotinib in Chronic Myeloid Leukemia Cells,” Blood 118 (2011): 2211–2221, 10.1182/blood-2010-10-313692. 21730355

[33] S. Melnik , D. Dvornikov , K. Müller‐Decker , et al., “Cancer Cell Specific Inhibition of Wnt/β‐Catenin Signaling by Forced Intracellular Acidification,” Cell Discovery 4 (2018): 37, 10.1038/s41421-018-0033-2. 29977599 PMC6028397

[34] S. Laurance , M. N. Aghourian , Z. Jiva Lila , C. A. Lemarie , and M. D. Blostein , “Gas6‐induced Tissue Factor Expression in Endothelial Cells Is Mediated through Caveolin‐1–Enriched Microdomains,” Journal of Thrombosis and Haemostasis 12 (2014): 395–408, 10.1111/jth.12481. 24354620

[35] C. Zhu , Y. Wei , and X. Wei , “AXL Receptor Tyrosine Kinase as a Promising Anti‐cancer Approach: Functions, Molecular Mechanisms and Clinical Applications,” Molecular Cancer 18 (2019): 153, 10.1186/s12943-019-1090-3. 31684958 PMC6827209

[36] Y. Jin , D. Nie , J. Li , et al., “Gas6/AXL Signaling Regulates Self‐Renewal of Chronic Myelogenous Leukemia Stem Cells by Stabilizing β‐Catenin,” Clinical Cancer Research 23 (2017): 2842–2855, 10.1158/1078-0432.CCR-16-1298. 27852702

[37] D. Lingwood and K. Simons , “Lipid Rafts as a Membrane‐organizing Principle,” Science 327 (2010): 46–50, 10.1126/science.1174621. 20044567

[38] B. Wang and P. Tontonoz , “Phospholipid Remodeling in Physiology and Disease,” Annual Review of Physiology 81 (2019): 165–188, 10.1146/annurev-physiol-020518-114444. PMC7008953 30379616

[39] T. Harayama , M. Eto , H. Shindou , et al., “Lysophospholipid Acyltransferases Mediate Phosphatidylcholine Diversification to Achieve the Physical Properties Required In Vivo,” Cell Metabolism 20 (2014): 295–305, 10.1016/j.cmet.2014.05.019. 24981836

[40] S. Akagi , N. Kono , H. Ariyama , H. Shindou , T. Shimizu , and H. Arai , “Lysophosphatidylcholine Acyltransferase 1 Protects against Cytotoxicity Induced by Polyunsaturated Fatty Acids,” The FASEB Journal 30 (2016): 2027–2039, 10.1096/fj.201500149. 26887439

[41] Z. Li , Y. Hu , H. Zheng , et al., “LPCAT1‐Mediated Membrane Phospholipid Remodelling Promotes Ferroptosis Evasion and Tumour Growth,” Nature Cell Biology 26 (2024): 811–824, 10.1038/s41556-024-01405-y. 38671262

[42] J. Bi , T.‐A. Ichu , C. Zanca , et al., “Oncogene Amplification in Growth Factor Signaling Pathways Renders Cancers Dependent on Membrane Lipid Remodeling,” Cell Metabolism 30 (2019): 525–538, 10.1016/j.cmet.2019.06.014. 31303424 PMC6742496

[43] J. A. Bennett , M. A. Mastrangelo , S. K. Ture , et al., “The Choline Transporter Slc44a2 Controls Platelet Activation and Thrombosis by Regulating Mitochondrial Function,” Nature Communications 11 (2020): 3479, 10.1038/s41467-020-17254-w. PMC7359028 32661250

[44] H. W. Kang , J. Wei , and D. E. Cohen , “PC‐TP/StARD2: of Membranes and Metabolism,” Trends in Endocrinology & Metabolism 21 (2010): 449–456, 10.1016/j.tem.2010.02.001. 20338778 PMC2897958

[45] A. Floris , J. Luo , J. Frank , et al., “Star‐related Lipid Transfer Protein 10 (STARD10): a Novel Key Player in Alcohol‐induced Breast Cancer Progression,” Journal of Experimental & Clinical Cancer Research 38 (2019): 4, 10.1186/s13046-018-1013-y. 30611309 PMC6321732

[46] R. Zhuo , Z. Zhang , Y. Chen , et al., “CDK5RAP3 is a Novel Super‐Enhancer‐Driven Gene Activated by Master TFs and Regulates ER‐Phagy in Neuroblastoma,” Cancer Letters 591 (2024): 216882, 10.1016/j.canlet.2024.216882. 38636893

[47] S. Xiong , J. Zhou , T. K. Tan , et al., “Super Enhancer Acquisition Drives Expression of Oncogenic PPP1R15B That Regulates Protein Homeostasis in Multiple Myeloma,” Nature Communications 15 (2024): 6810, 10.1038/s41467-024-50910-z. PMC11316114 39122682

[48] M. Furlan‐Magaril , H. Rincon‐Arano , and F. Recillas‐Targa , “Sequential Chromatin Immunoprecipitation Protocol: ChIP‐reChIP,” Methods in Molecular Biology 543 (2009): 253–266. 19378171 10.1007/978-1-60327-015-1_17

[49] B. Zhu , W. Zhong , X. Cao , et al., “Loss of miR‐31‐5p Drives Hematopoietic Stem Cell Malignant Transformation and Restoration Eliminates Leukemia Stem Cells in Mice,” Science Translational Medicine 14 (2022): abh2548, 10.1126/scitranslmed.abh2548. 35080912

[50] C. Peng and S. Li , “Chronic Myeloid Leukemia (CML) Mouse Model in Translational Research,” Methods in Molecular Biology 1438 (2016): 225–243. 27150093 10.1007/978-1-4939-3661-8_13

