# Supplementary material for: Super‐Enhancer‐Driven SOX4/SMAD3 Mediate Membrane Remodeling by Regulating Phospholipid Metabolism to Accelerate Leukemia Progression
Source: Adv Sci (Weinh). 2026 Feb 21;13(24):e12332. doi: 10.1002/advs.202512332 (PMC13116350; doi:10.1002/advs.202512332)
Supplement: Supplementary file 1 — Supporting File 1: advs74452‐sup‐0001‐SuppMat.docx. [file ADVS-13-e12332-s001.docx]

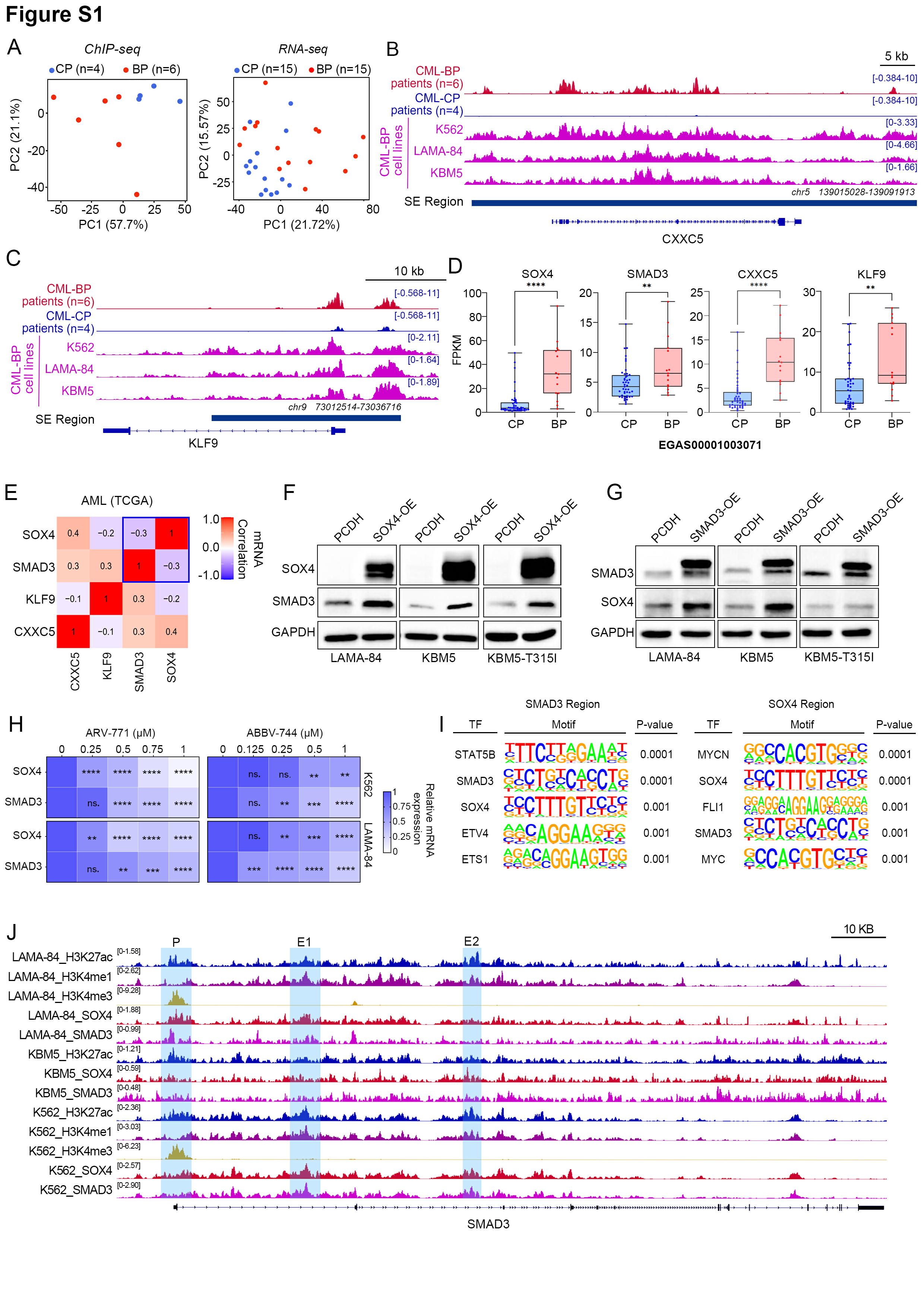


**Figure S1**

(A) PCA of ChIP-seq and RNA-seq data of CML-CP and BP patient samples.

(B-C) IGV tracks of H3K27ac ChIP-seq signals at the genomic loci of CXXC5 (B) and KLF9 (C) in CML-CP patients, CML-BP patients, and CML-BP cell lines.

(D)The expression changes of SOX4 and SMAD3 between CML-CP and CML-BP (EGAS00001003071).

(E) Pearson correlation analysis of candidate SE-driven TFs in AML (TCGA).

(F) Western blot analysis of SOX4 and SMAD3 in LAMA-84, KBM5 and KBM5-T315I cells transfected with SOX4 overexpression vector or empty vector.

(G) Western blot analysis of SOX4 and SMAD3 in LAMA-84, KBM5 and KBM5-T315I cells transfected with SMAD3 overexpression vector or empty vector.

(H) mRNA expression levels of SOX4 and SMAD3 in CML-BP cells treated with ARV771 or ABBV-744.

(I) Motif enrichment analysis in the genomic regions of SOX4 and SMAD3.

(J) Representative IGV tracks showing co-occupancy of SOX4 and SMAD3 at the SMAD3 gene locus in CML-BP cells. Two candidate enhancer regions (E1, E2) and one promoter region (P) are indicated.


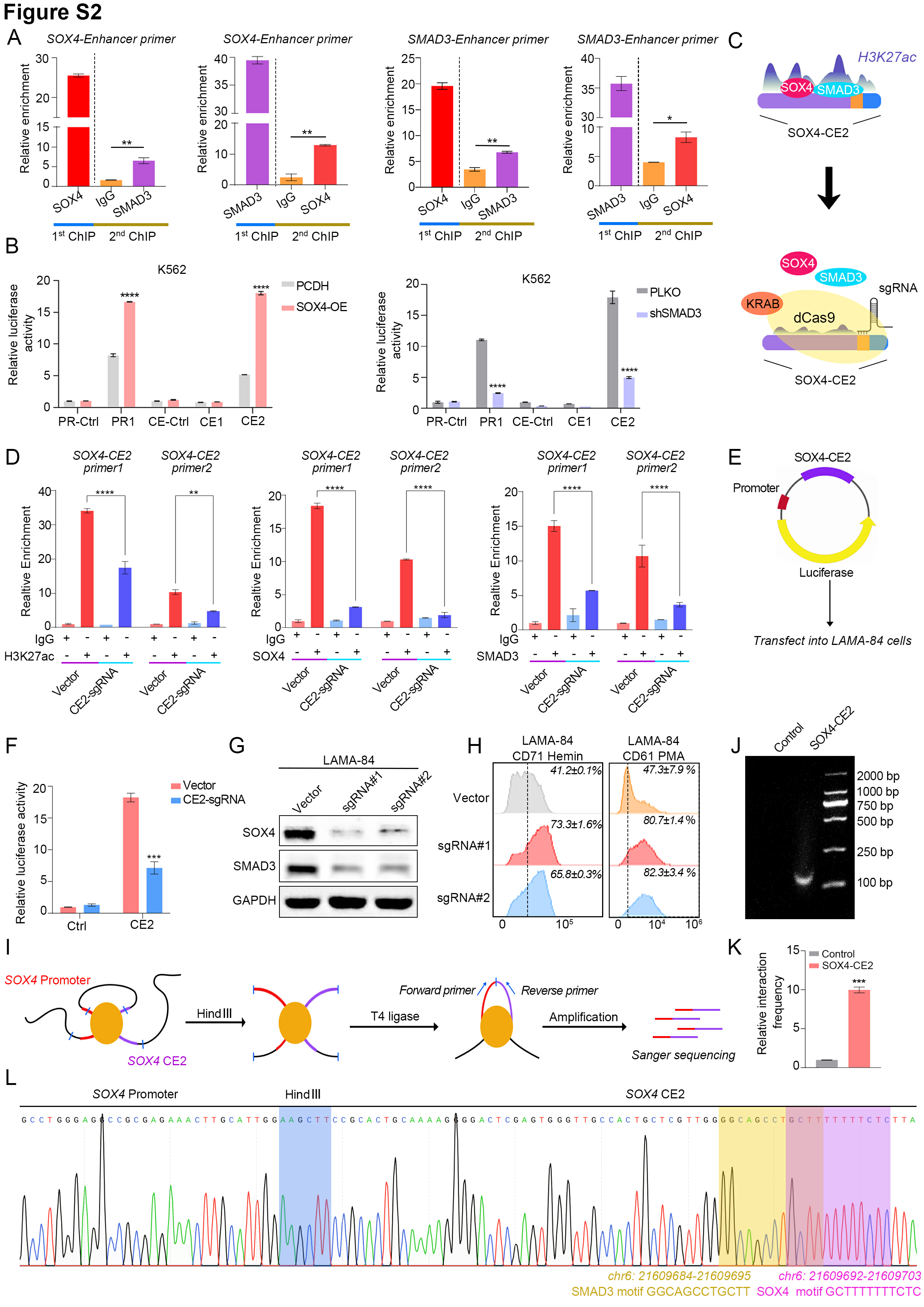


**Figure S2**

(A) ChIP-re-ChIP showing co-occupancy of SOX4 and SMAD3 on SOX4-Enhancer and SMAD3-Enhancer. ChIP products immunoprecipitated by each of the two antibodies were subjected to re-ChIP using the another antibody or normal immunoglobulin.

(B) Luciferase reporter assays measuring promoter and enhancer activities in K562 cells under SOX4 overexpression or knockdown conditions compared to respective controls.

(C) Schematic illustration of the dCas9-KRAB system for targeted SOX4-CE2.

(D) ChIP-qPCR targeting SOX4-CE2 was performed on LAMA-84 cells transfected vector or sgRNA targeting SOX4-CE2 using indicated antibody. IgG was used as a negative control antibody.

(E) Schematic diagram of the non-sgRNA-targeted SOX4-CE2 region cloned into the pGL3-Promoter.

(F) Non-sgRNA-targeted SOX4-CE2 activities were measured by luciferase reporter assays in LAMA-84 cells with the indicated sgRNA or vector.

(G) Western blot detect the protein expression of SOX4 and SMAD3 in the LAMA-84 cells transfected vector or sgRNAs targeting SOX4-CE2.

(H) Representative flow cytometry histogram of surface CD71 and CD61 expression in LAMA-84 cells transfected with CE2-targeting sgRNAs.

(I) Workflow schematic of the chromosome conformation capture (3C) assay procedure.

(J) PCR-agarose gel electrophoresis analysis of 3C ligation products testing interactions between SOX4-CE2 and SOX4 promoter regions in LAMA-84 cells.

(K) Quantitative 3C analysis measuring contact frequencies between CE2 and promoter regions within the SOX4 chromatin loop domain in LAMA-84 cells.

(L) Sanger sequencing results from 3C products verifying the specific physical interaction between CE2 and SOX4 promoter regions.


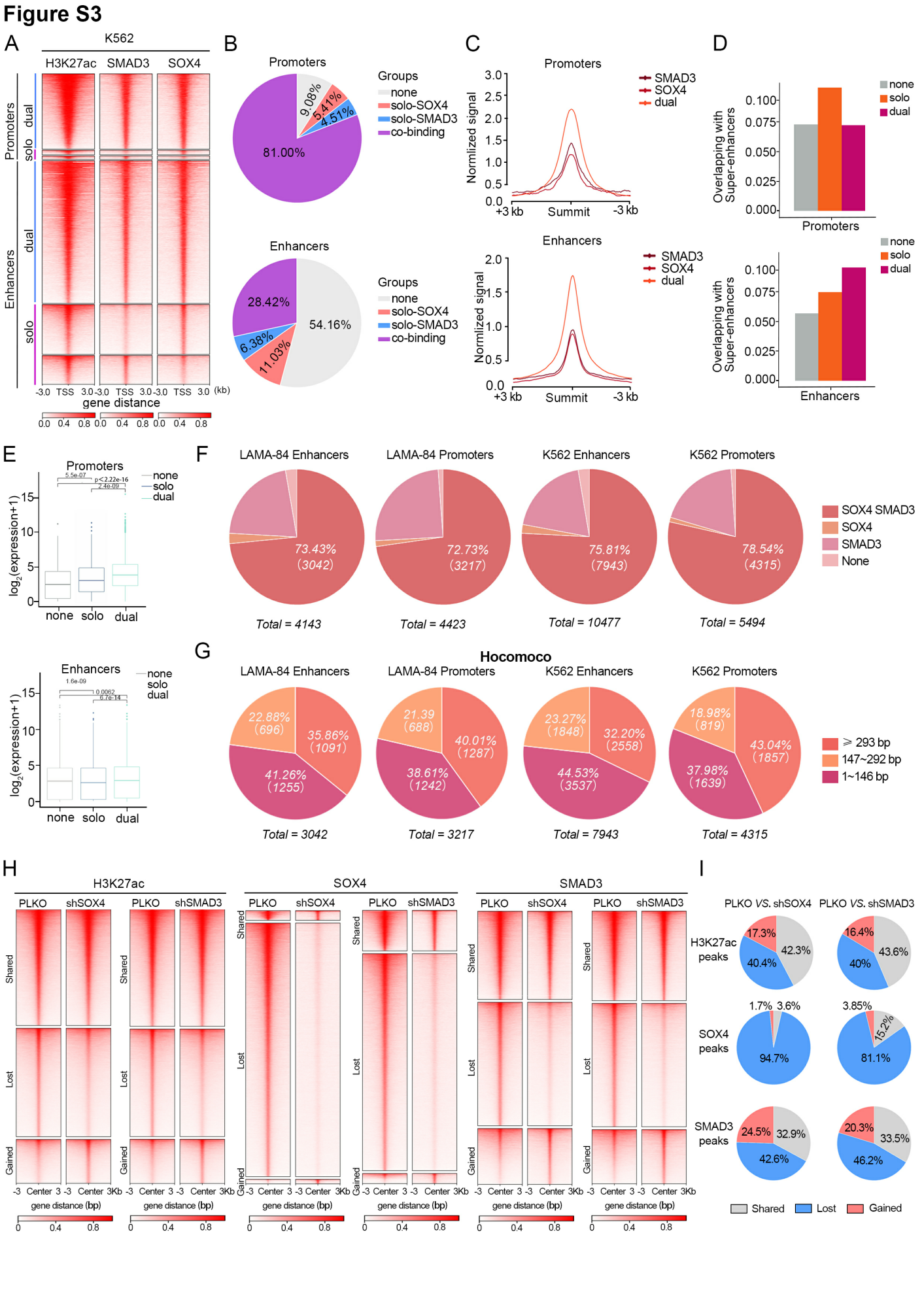


**Figure S3**

(A)Heatmaps displaying CUT&Tag signals for SOX4, SMAD3, and H3K27ac in K562 cells, centered on peak summits and grouped by distinct SOX4/SMAD3 co-binding patterns (SOX4-only, SMAD3-only, dual-bound).

(B) Pie chart quantification of the genome-wide distribution of SOX4 and SMAD3 binding patterns in K562 cells, showing the relative proportions of solo- and co-bound regions.

(C) Line plots of H3K27ac CUT & Tag signals from indicated groups of peaks in K562 cells.

(D) The overlapping of indicated groups of peaks with super-enhancers in K562 cells.

(E) Box plots of mRNA expression of genes regulated by the indicated groups of peaks in LAMA-84 cells.

(F) Pie chart analysis of motif occurrence in SOX4/SMAD3 co-bound peak regions in K562 and LAMA-84 cells, showing the proportion of regions containing both SOX4 and SMAD3 motifs, SOX4 motif only, SMAD3 motif only, or neither motif.

(G) Spatial distribution analysis of SOX4 and SMAD3 binding motifs within their co-occupied genomic regions using Hocomoco.

(H) Heatmaps displaying H3K27ac, SOX4, and SMAD3 CUT&Tag signals between control (PLKO) and individual TF-knockdown groups in LAMA-84 cells.

(I) Pie charts showing the proportions of gained, lost, or unchanged peaks for H3K27ac, SOX4, and SMAD3 separately upon transcription factor knockdown in LAMA-84 cells.


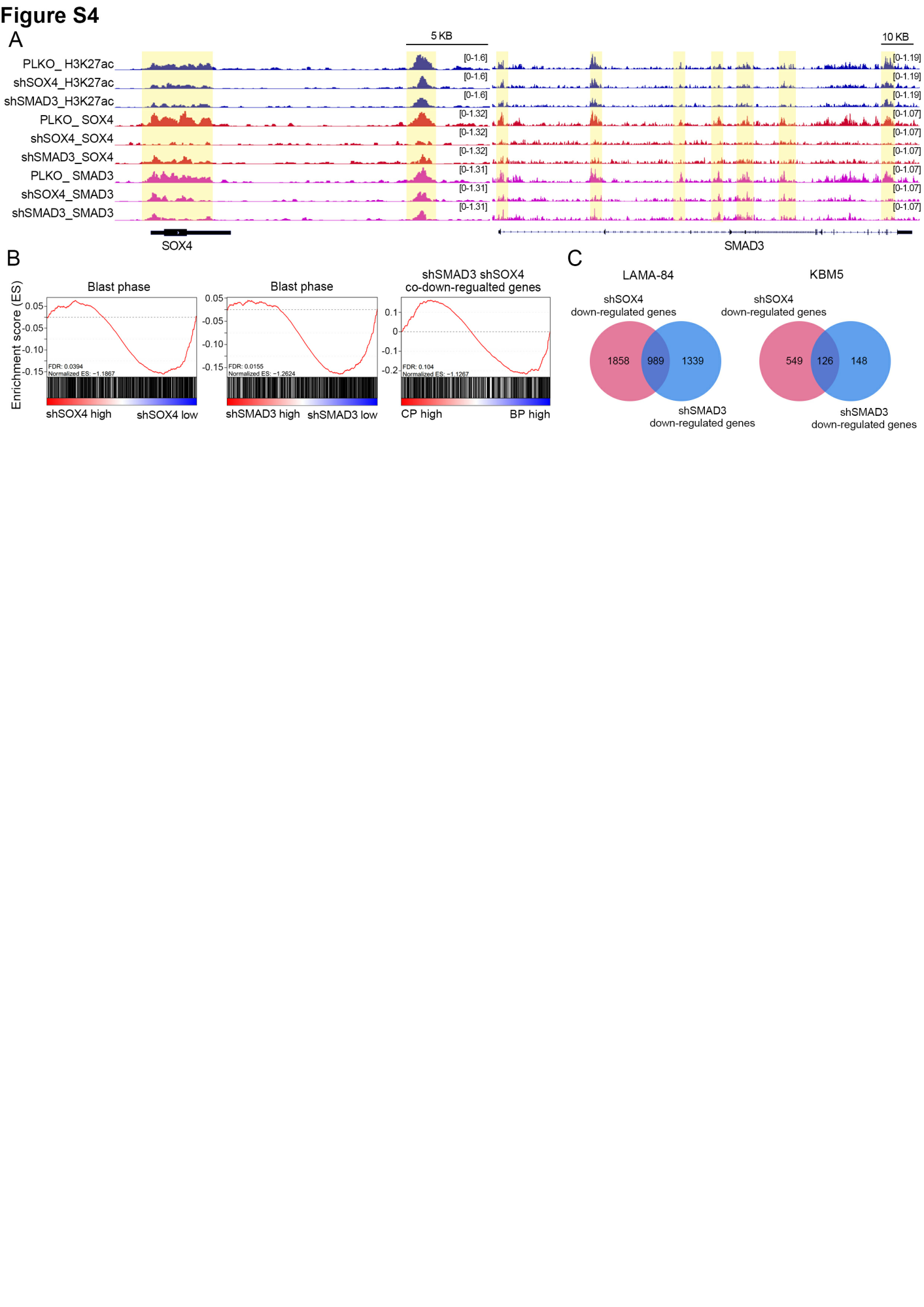


**Figure S4**

(A) IGV tracks displaying SOX4, SMAD3, and H3K27ac binding signals at the SOX4 and SMAD3 gene loci in LAMA-84 control (PLKO), shSOX4, and shSMAD3 groups.

(B) GSEA analysis showing enrichment of blast phase gene signatures in: (left) genes downregulated by SOX4 knockdown; (middle) genes downregulated by SMAD3 knockdown; (right) genes co-downregulated by both SOX4 and SMAD3 knockdown in the BP-highly-expressed gene set (EGAS00001003071).

(C) Venn diagram identifying the shared downregulated genes following SOX4 or SMAD3 knockdown in both LAMA-84 and KBM5 cells.


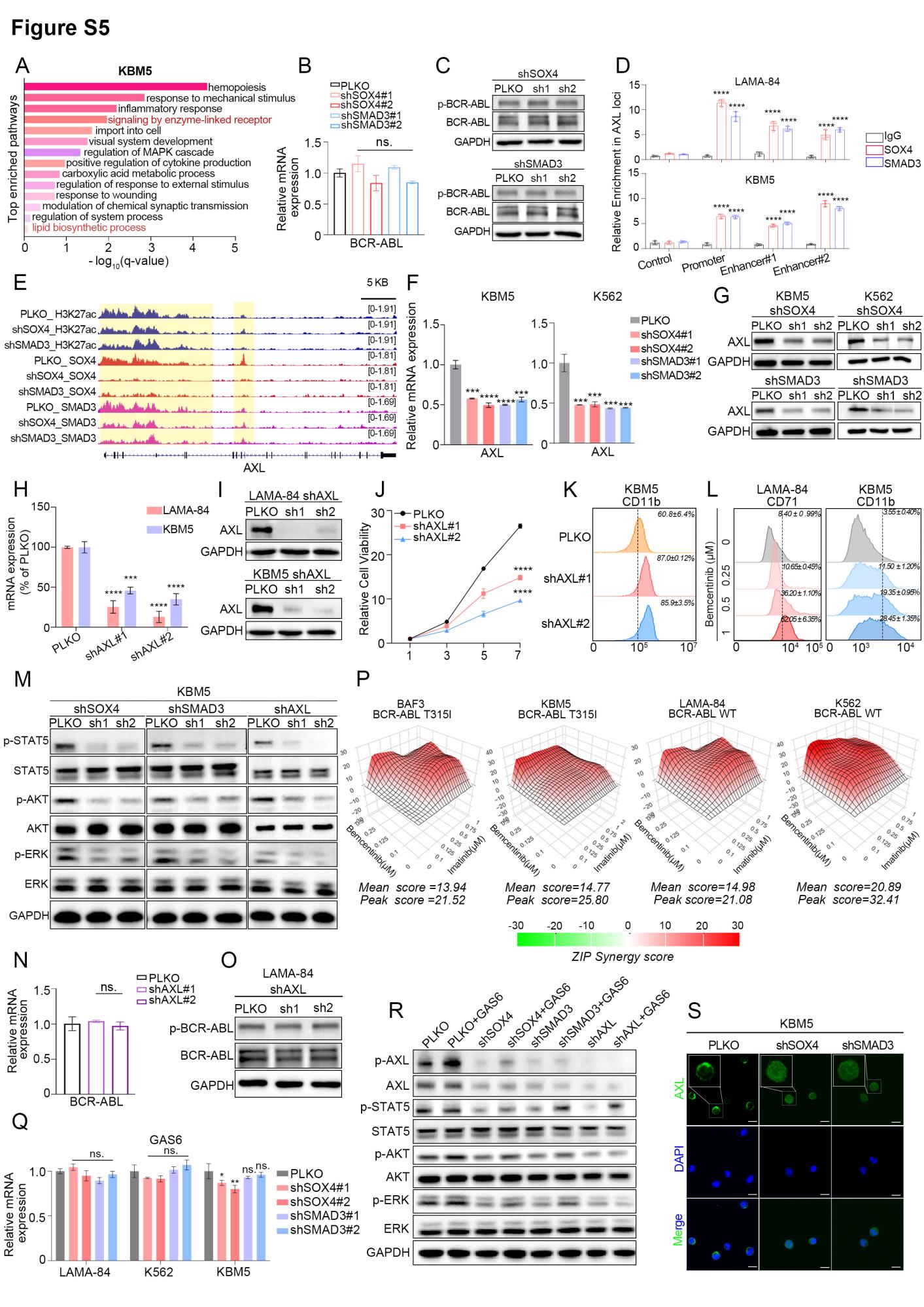


**Figure S5**

(A)Top GO pathway enrichment analysis of the shared downregulated genes in KBM5 cells following SOX4 or SMAD3 knockdown.

(B-C) RT-qPCR (B) and Western blot (C) analysis of BCR-ABL mRNA and protein levels in LAMA-84 cells following SOX4 or SMAD3 knockdown.

(D) CUT&Tag-qPCR analysis showing the enrichment of SOX4 and SMAD3, relative to control IgG, at the promoter and enhancer regions of the AXL gene in KBM5 and LAMA-84 cells.

(E) IGV tracks displaying SOX4, SMAD3, and H3K27ac binding signals at the AXL locus in LAMA-84 control (PLKO), shSOX4, and shSMAD3 groups.

(F-G) RT-qPCR (F) and Western blot (G) analysis of AXL expression following SOX4 or SMAD3 knockdown in KBM5 and LAMA-84 cells following SOX4 or SMAD3 knockdown.

(H-I) Knockdown efficiency validation of AXL shRNAs at mRNA (H) and protein (I) level in LAMA-84 and KBM5 cells.

(J) The relative cell viability of KBM5 cells expressing empty vector and shAXL.

(K) Flow cytometry analysis of granulocytic differentiation marker CD11b in KBM5 cells with AXL knockdown following ATRA treatment.

(L) Flow cytometry analysis of surface expression of CD71 (left) and CD11b (right) in CML-BP cells following Bemcentinib treatment.

(M) Western blot analysis of AKT, STAT5, and ERK phosphorylation in KBM5 cells following SOX4, SMAD3, or AXL knockdown.

(N-O) RT-qPCR (N) and Western blot (O) analysis analysis of BCR-ABL levels in LAMA-84 cells with AXL knockdown.

(P) Synergy assessment between imatinib and Bemcentinib using Zero Interaction Potency (ZIP) model in CML cell lines after 48-hour treatment.

(Q) RT-qPCR analysis of GAS6 expression in CML-BP cells following SOX4 ,SMAD3 or AXL knockdown.

(R) Western blot analysis of AKT, STAT5, and ERK phosphorylation in LAMA-84 cells with indicated groups, with or without GAS6 stimulation.

(S) Immunofluorescence staining of AXL localization (green) in KBM5 cells with indicated shRNA or vector. Nuclei were stained with DAPI (blue). Scale bar, 10 μm.


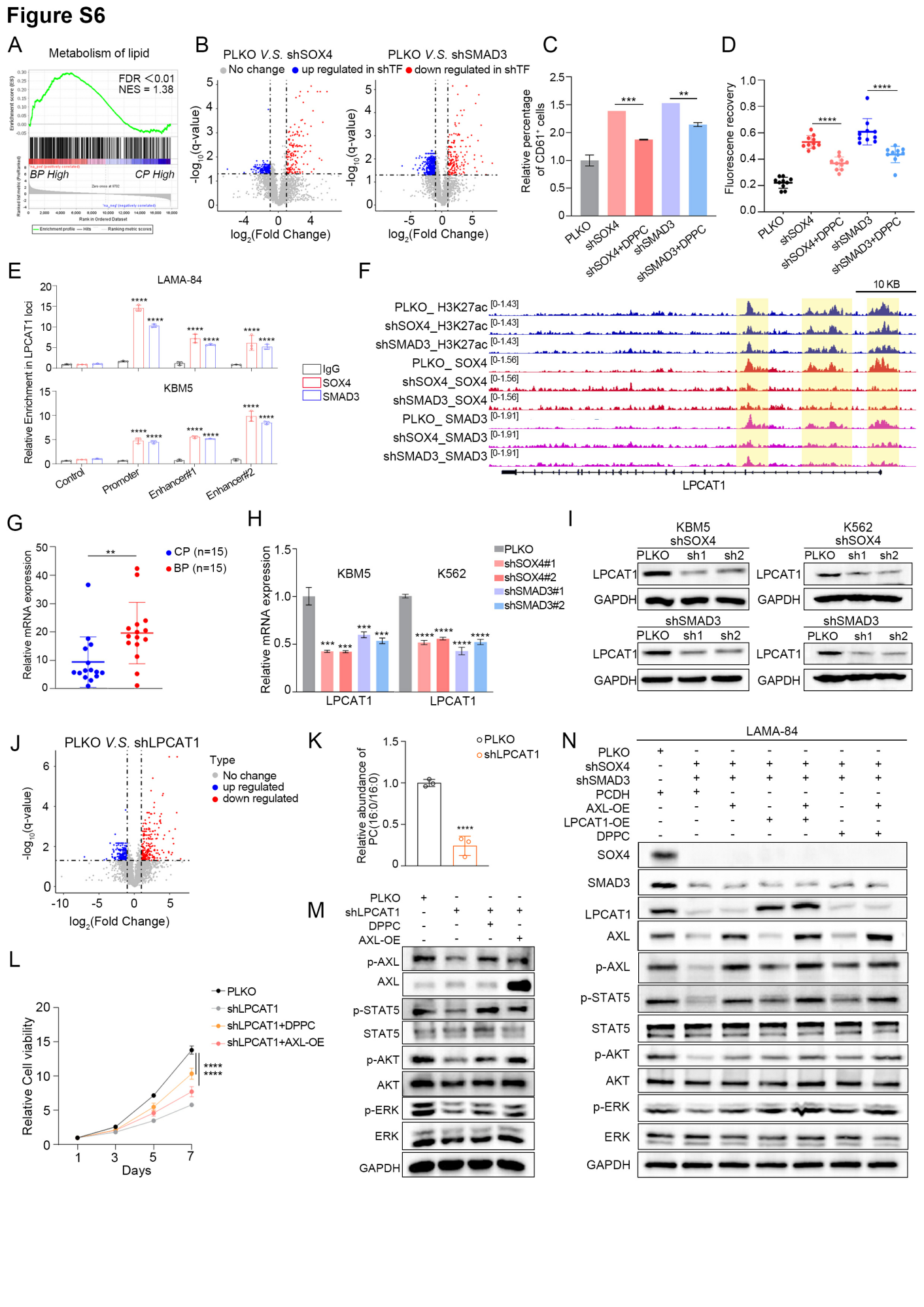


**Figure S6**

(A) GSEA shows that gene sets related to lipid metabolism are significantly enriched in the gene expression profile of CML-BP.

(B) Volcano plots of lipidomic analysis showing differentially expressed lipid ions upon SOX4/SMAD3 knockdown in LAMA-84 cells. Each dot represents an individual lipid ion.

(C) Representative flow cytometry analysis of surface CD61 expression in LAMA-84 cells with indicated shRNA or empty vector, with or without DPPC liposome supplementation.

(D) Quantification of total fluorescence recovery in FRAP assays following photobleaching in LAMA-84 cells with indicated shRNA or empty vector, with or without DPPC liposome supplementation. n=10.

(E) CUT&Tag-qPCR analysis showing the enrichment of SOX4 and SMAD3, relative to control IgG, at the promoter and enhancer regions of the LPCAT1 gene in KBM5 and LAMA-84 cells.

(F) IGV tracks showing SOX4, SMAD3, and H3K27ac binding signals at the LPCAT1 loci in LAMA-84 control (PLKO), shSOX4, and shSMAD3 groups.

(G) LPCAT1 mRNA expression levels in primary patient samples of CML-CP (n=15) versus CML-BP (n=15).

(H) RT-qPCR analysis of LPCAT1 mRNA expression in KBM5 and K562 cells transfected with shRNA targeting SOX4, SMAD3, or empty vector control.

(I) Western blot analysis of LPCAT1 protein expression in KBM5 and K562 cells transfected with shRNA targeting SOX4, SMAD3, or empty vector control.

(J) Volcano plots of lipidomic analysis showing differentially expressed lipid ions upon LPCAT1 knockdown in LAMA-84 cells. Each dot represents an individual lipid ion.

(K) Quantitative analysis of PC (16:0/16:0) (DPPC) abundance in LAMA-84 cells expressing LPCAT1 shRNA or empty vector control.

(L) Rescue of cell viability of LPCAT1-deficient LAMA-84 cells by DPPC supplementation or AXL cDNA overexpression.

(M) Western blot analysis of total and phosphorylation levels of indicated proteins in LPCAT1-deficient LAMA-84 cells rescued AXL cDNA overexpression or DPPC supplementation .

(N) Western blot analysis assessing transfection efficiency of LPCAT1 and AXL cDNA constructs, along with AXL-mediated downstream signaling in SOX4/SMAD3-deficient LAMA-84 cells (shTFs).


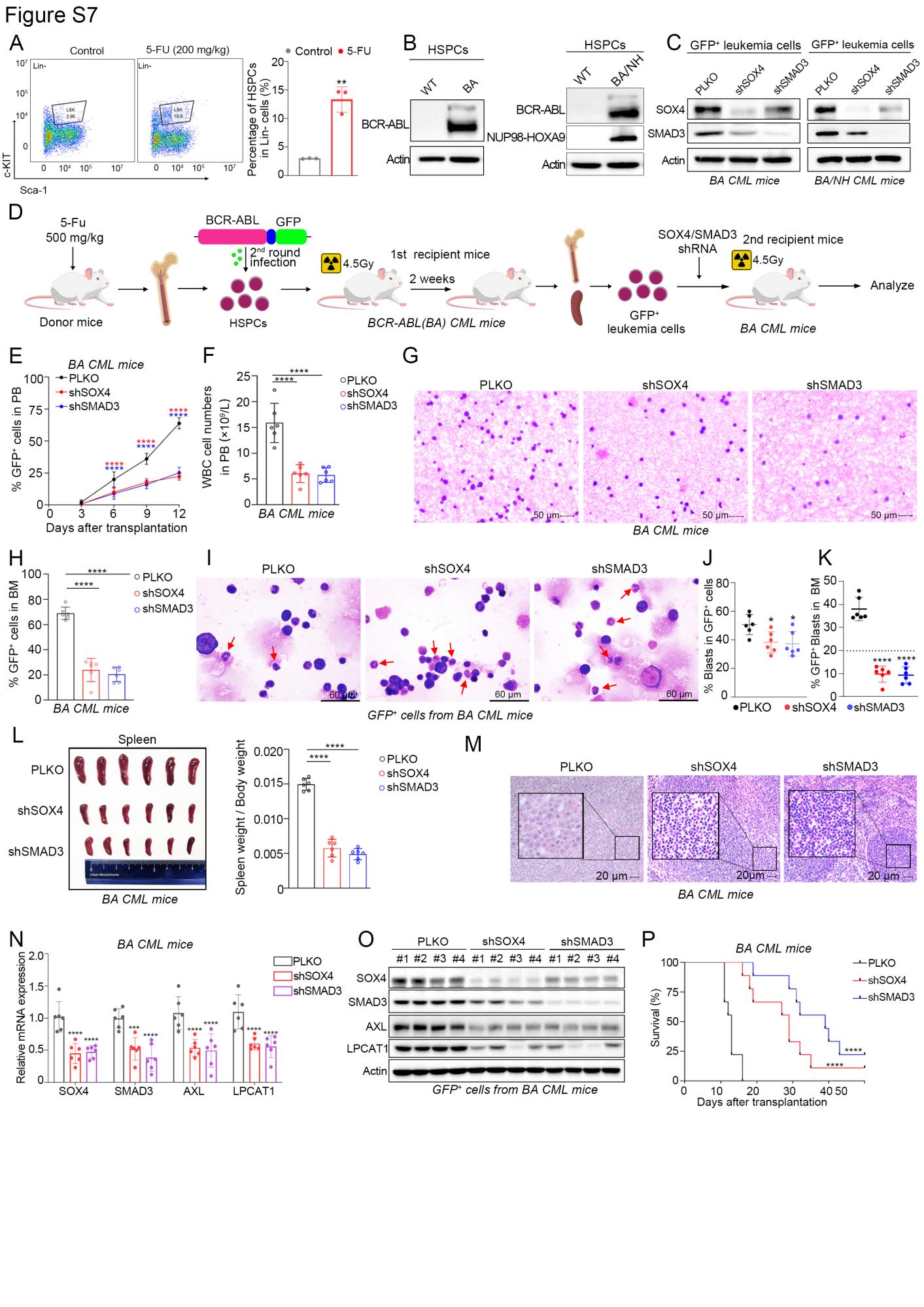


**Figure S7**

(A) Representative flow cytometry plot showing the gating strategy for sorting HSPCs (Lin⁻Sca-1⁺c-Kit⁺) from donor mouse bone marrow (Left). Quantification of HSPCs within the Lin⁻ bone marrow cell population from donor mice, n=3 (Right).

(B) Western blot analysis confirming BCR-ABL and NUP98-HOXA9 protein expression efficiency in HSPCs following retroviral transduction.

(C) Knockdown efficiency validation of SOX4 and SMAD3 shRNAs in GFP⁺ leukemia cells from primary CML mice by Western blot analysis.

(D) Schematic of the BCR-ABL(BA)-driven CML mouse model establishment and intervention strategy.

(E) The percentage of GFP^+^ cells in the PB of BA CML mice with SOX4/SMAD3 knockdown. n=6 mice per group.

(F) Total number of WBCs in PB from BA mice transplanted with vector or SOX4/SMAD3 shRNA-transduced leukemia cells. n=6 mice per group.

(G) Representative Wright-Giemsa staining of PB smears from BA CML mice transplanted with vector or SOX4/SMAD3 shRNA-transduced leukemia cells. n=6 mice per group.

(H) Percentages of GFP^+^ cells in BM from BA CML mice of the SOX4/SMAD3 knockdown group. n=6 mice per group.

(I) Representative Wright-Giemsa staining of BM smears prepared from flow-sorted GFP⁺ cells from BA CML mice of the SOX4/SMAD3 knockdown group. n=6 mice per group.

(J) The proportion of blast cells was analyzed in bone marrow smears prepared from sorted GFP^+^ cells following SOX4/SMAD3 knockdown in BA CML mice.

(K) The proportion of GFP^+^ blast cells was analyzed in bone marrow cells from BA CML mice with SOX4/SMAD3 knockdown.

(L) Gross pathology (left) and relative weights (right) of the spleens from BA CML mice of the SOX4/SMAD3 knockdown group. n=6 mice per group.

(M) Hematoxylin-eosin staining of the spleens from BA CML mice with SOX4 or SMAD3 knockdown. n=6 mice per group.

(N) RT-qPCR analysis of SOX4, SMAD3, AXL, and LPCAT1 mRNA expression in sorted GFP^+^ leukemic cells from BA CML mice of the control and knockdown groups. n=6 mice per group.

(O) Western blot analysis of SOX4, SMAD3, AXL, and LPCAT1 protein expression in leukemia cells from BA CML mice. n=4 mice per group.

(P) Kaplan-Meier survival curves of BA mice transplanted with vector or SOX4/SMAD3 shRNA-transduced leukemia cells. n=9 mice per group.


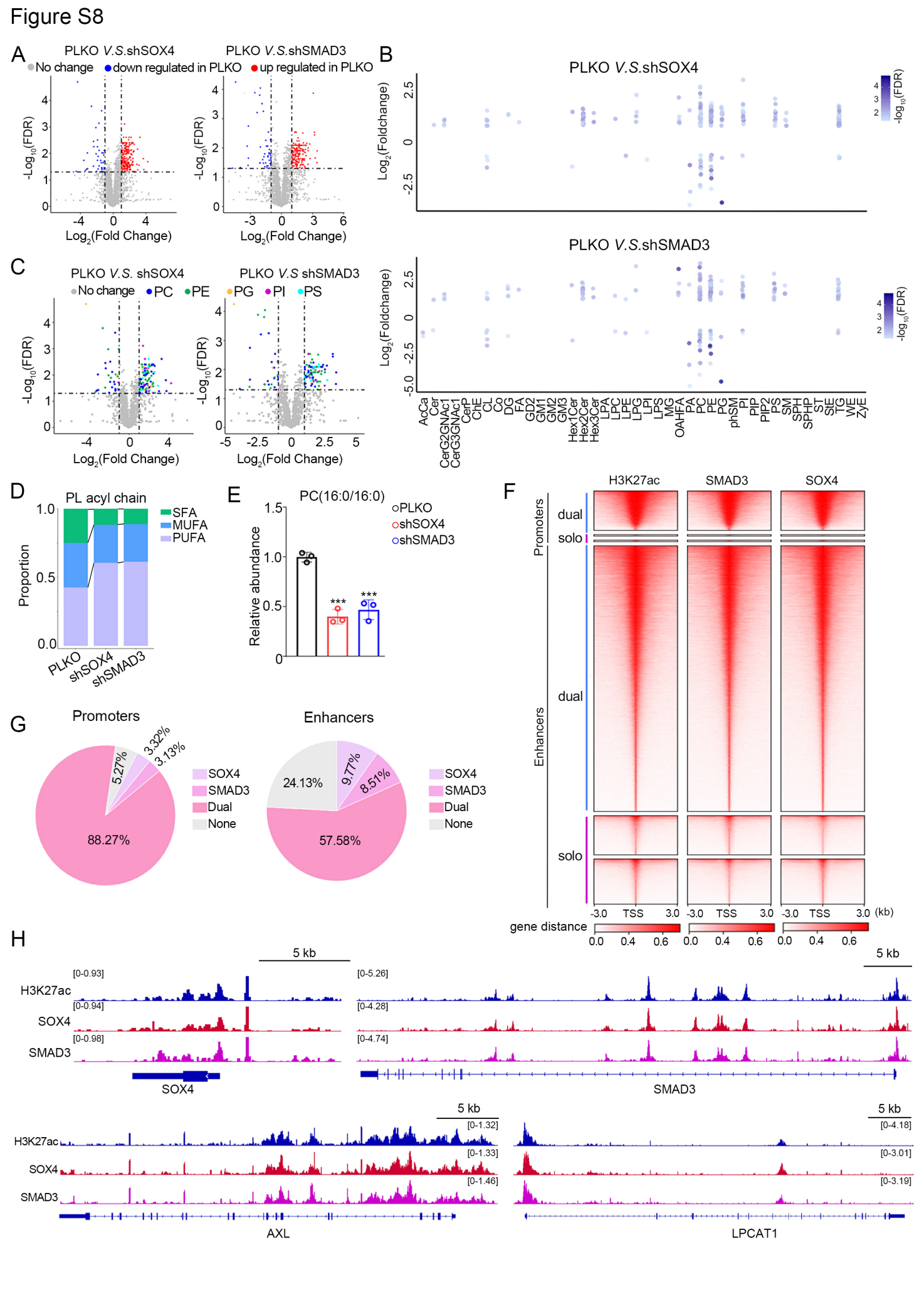


**Figure S8**

(A) Volcano plots showing differentially expressed lipid ions in leukemic cells from BA CML mice following SOX4 or SMAD3 knockdown compared to controls. Each dot represents a lipid ion.

(B) Comprehensive lipid subclass distribution analysis showing alterations in major lipid categories in leukemic cells from BA CML mice following SOX4 or SMAD3 knockdown.

(C) Volcano plots specifically highlighting differentially regulated phospholipid species in leukemic cells from BA CML mice of the SOX4- or SMAD3-knockdown groups.

(D) The repartition of PL-SFAs, PL-MUFAs and PL-PUFAs in leukemic cells from BA CML mice with SOX4 or SMAD3 knockdown.

(E) Quantitative analysis of PC (16:0/16:0) (DPPC) abundance in leukemic cells from BA CML mice following SOX4 or SMAD3 knockdown.

(F) Heatmaps displaying CUT&Tag signals for SOX4, SMAD3, and H3K27ac in leukemic cells from BA CML mice, centered on peak summits and grouped by distinct SOX4/SMAD3 co-binding patterns (SOX4-only, SMAD3-only, dual-bound).

(G) Pie chart quantification of the genome-wide distribution of SOX4 and SMAD3 binding patterns in leukemic cells from BA CML mice, showing the relative proportions of solo- and co-bound regions.

(H) IGV tracks showing SOX4 and SMAD3 binding patterns at the SOX4, SMAD3, AXL, and LPCAT1 genomic loci in leukemic cells from the BA CML mouse model.


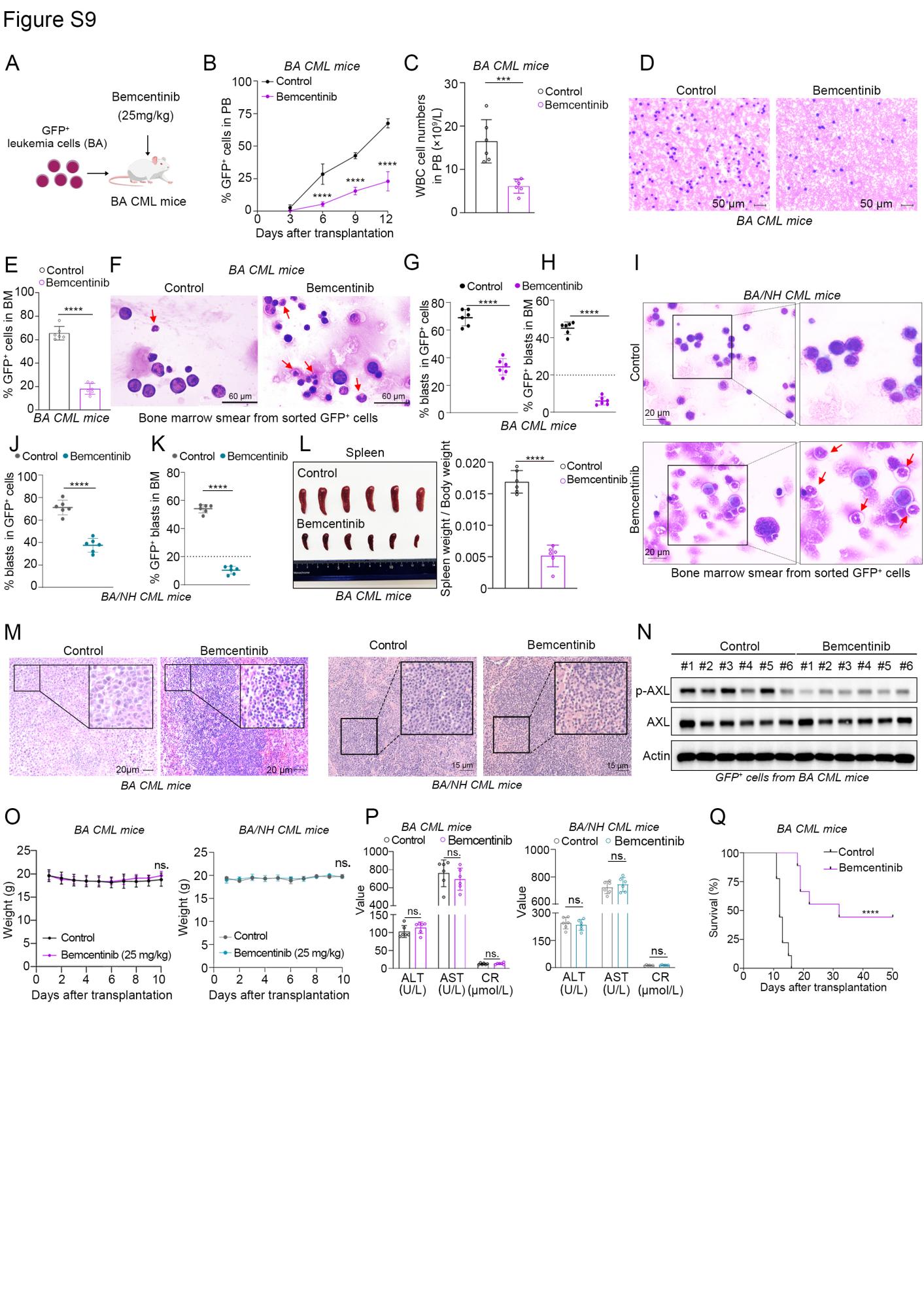


**Figure S9**

(A) Experimental schema of Bemcentinib treatment in the BA CML mouse model.

(B) The percentage of GFP^+^ cells in the PB of BA CML mice with Bemcentinib-treated. n=6 mice per group.

(C) Total number of WBCs in the PB of CML mice with Bemcentinib-treated. n=6 mice per group.

(D) Wright-Giemsa staining of PB smears of BA CML mice with Bemcentinib-treated. n=6 mice per group.

(E) The percentage of GFP^+^ cells in BM of BA CML mice with Bemcentinib-treated. n=6 mice per group.

(F) Wright-Giemsa staining of bone marrow smears prepared from sorted GFP^+^ cells in Bemcentinib-treated BA CML mice.

(G) The proportion of blast cells was analyzed in bone marrow smears prepared from sorted GFP+ cells following Bemcentinib-treated in BA CML mice.

(H) The proportion of GFP+ blast cells was analyzed in bone marrow cells from BA CML mice with Bemcentinib-treated.

(I) Wright-Giemsa staining of bone marrow smears prepared from sorted GFP^+^ cells in Bemcentinib-treated BA/NH CML mice.

(J) The proportion of blast cells was analyzed in bone marrow smears prepared from sorted GFP+ cells following Bemcentinib-treated in BA/NH CML mice.

(K) The proportion of GFP+ blast cells was analyzed in bone marrow cells from BA/NH CML mice with Bemcentinib-treated.

(L) Gross pathology (left) and relative weights (right) of the spleens from BA CML mice with Bemcentinib-treated. n=6 mice per group.

(M)Hematoxylin-eosin staining of the spleens from BA and BA/NH CML mice with Bemcentinib-treated. n=6 mice per group.\

(N) Western blot analysis of the total and phosphorylation levels of AXL in leukemia cells from BA CML mice. n=6 mice per group.

(O) Body weight monitoring of BA and BA/NH CML mice during Bemcentinib treatment course.

(P) Serum biochemical analysis for treatment safety assessment. Alanine aminotransferase (ALT), aspartate aminotransferase (AST), and creatinine (CR) levels in Bemcentinib-treated BA and BA/NH mice. n=6 mice per group.

(Q) Kaplan-Meier survival curves of Bemcentinib-treated versus vehicle-treated BA CML mice. n=9 mice per group.
